# Supplementary material for: Protein-Protein Binding as a Two-Step Mechanism: Preselection of Encounter Poses during the Binding of BPTI and Trypsin
Source: Biophys J. 2020 Jul 10;119(3):652–66. doi: 10.1016/j.bpj.2020.06.032 (PMC7399559; doi:10.1016/j.bpj.2020.06.032)
Supplement: Document S1. Figures S1–S17 and Table S1 [file mmc1.pdf]

Biophysical Journal, Volume *119*

Supplemental Information

Protein-Protein Binding as a Two-Step Mechanism: Preselection of Encounter Poses during the Binding of BPTI and Trypsin

Ursula Kahler, Anna S. Kamenik, Franz Waibl, Johannes Kraml, and Klaus R. Liedl

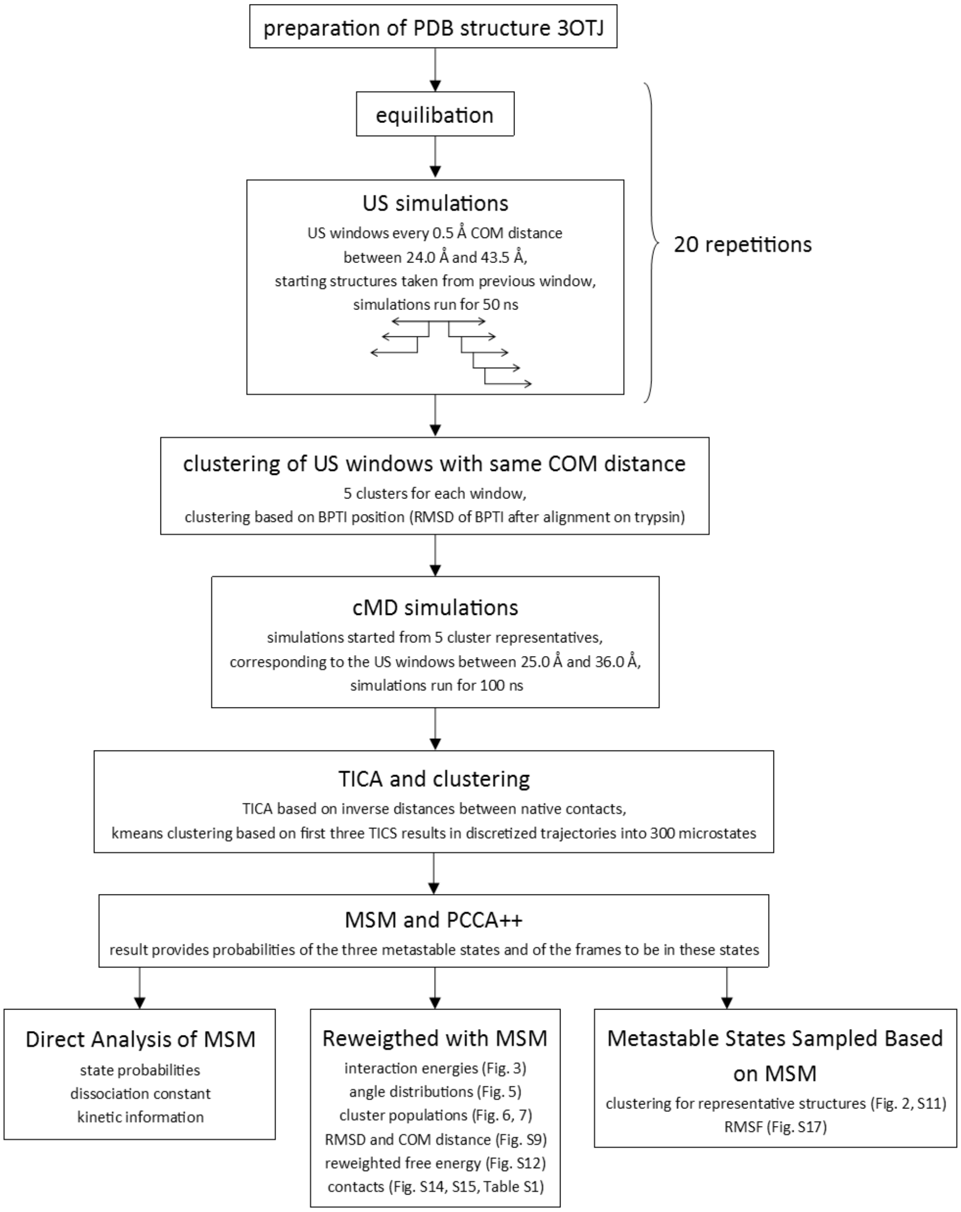

Figure S1: Workflow applied in the preparation of this paper. After preparation and equilibation of a crystal structure of the Trypsin–BPTI complex, twenty separate US simulations are used to sample the unbinding. As these cover the whole pathway, it is expected that using them to seed cMD simulations strongly enhances the sampling efficiency. Based on the cMD simulations, an MSM is build following the typical workflow, including coordinate reduction and discretization of the trajectories. The MSM determines the classification into three metastable states and builds the foundation for the subsequent analyses.

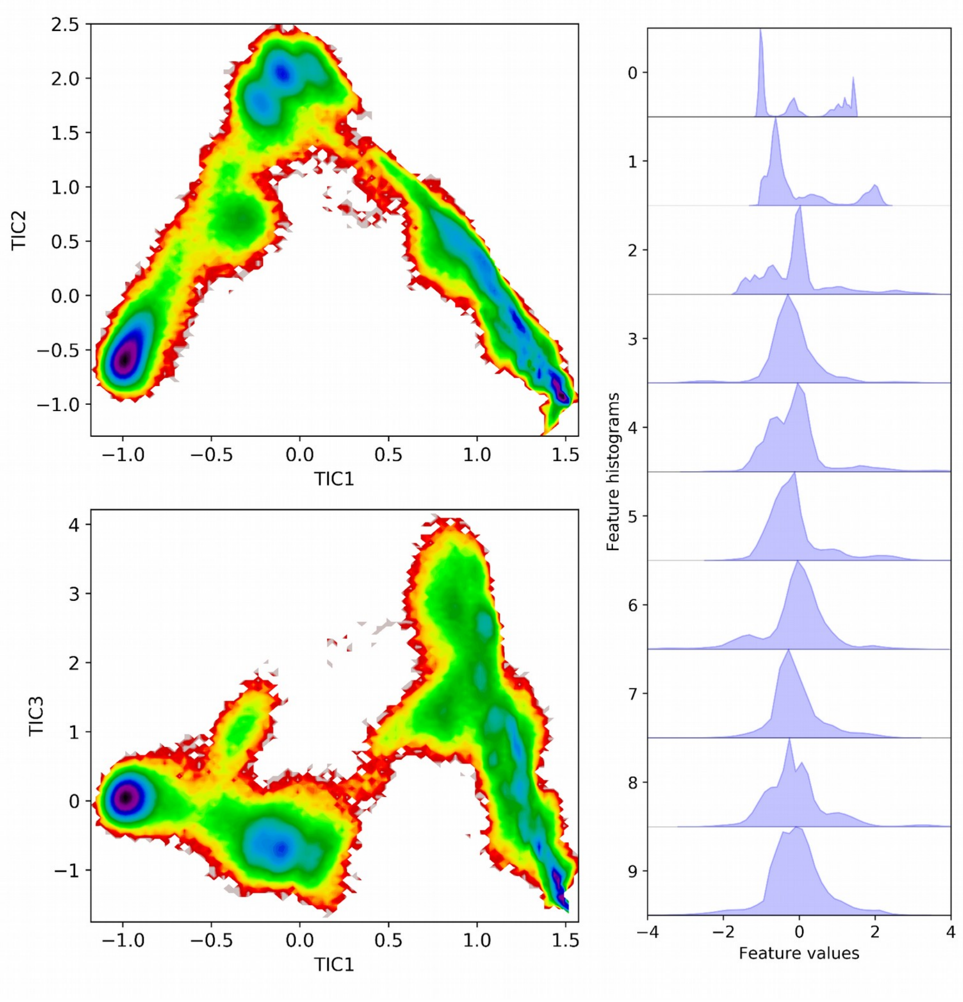

Figure S2: Projection of the simulation frames on the TICs. Top left: Density of the frames within TIC1 and TIC2; bottom left: density of the frames within TIC1 and TIC3; right: distribution of the frames within the first ten TICs.

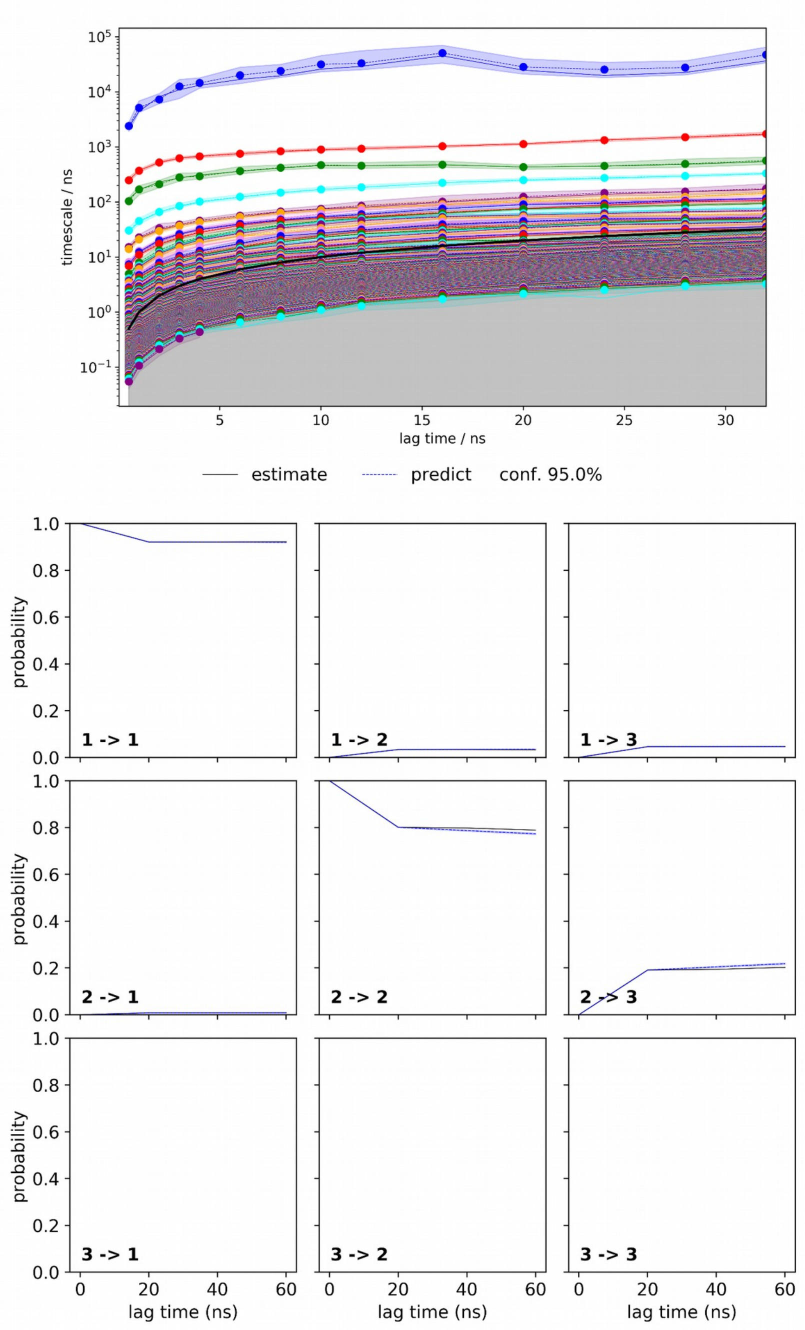

Figure S3: Chapman-Kolmogorov tests of the MSM. Top: The implied timescale plot helps to choose the lag time, for which the calculated timescales are constant. We chose a lag time of 20 ns although the loss of connectivity causes deviations from the ideal behavior. Bottom: The plots show the result of the Chapman-Kolmogorov test for 20, 40 and 60 ns, comparing the results predicted by the used MSM (lag time of 20 ns, dashed line) and estimated at the different lag times (continuous line). In some panels, both lines can be hardly seen, as they run along the 0- (2 -> 1, 3 -> 1 and 3 -> 2) or 1-line (3 -> 3) of the plots. Thereby, 1 denotes the unbound state, 2 the encounter state and 3 the complex state.

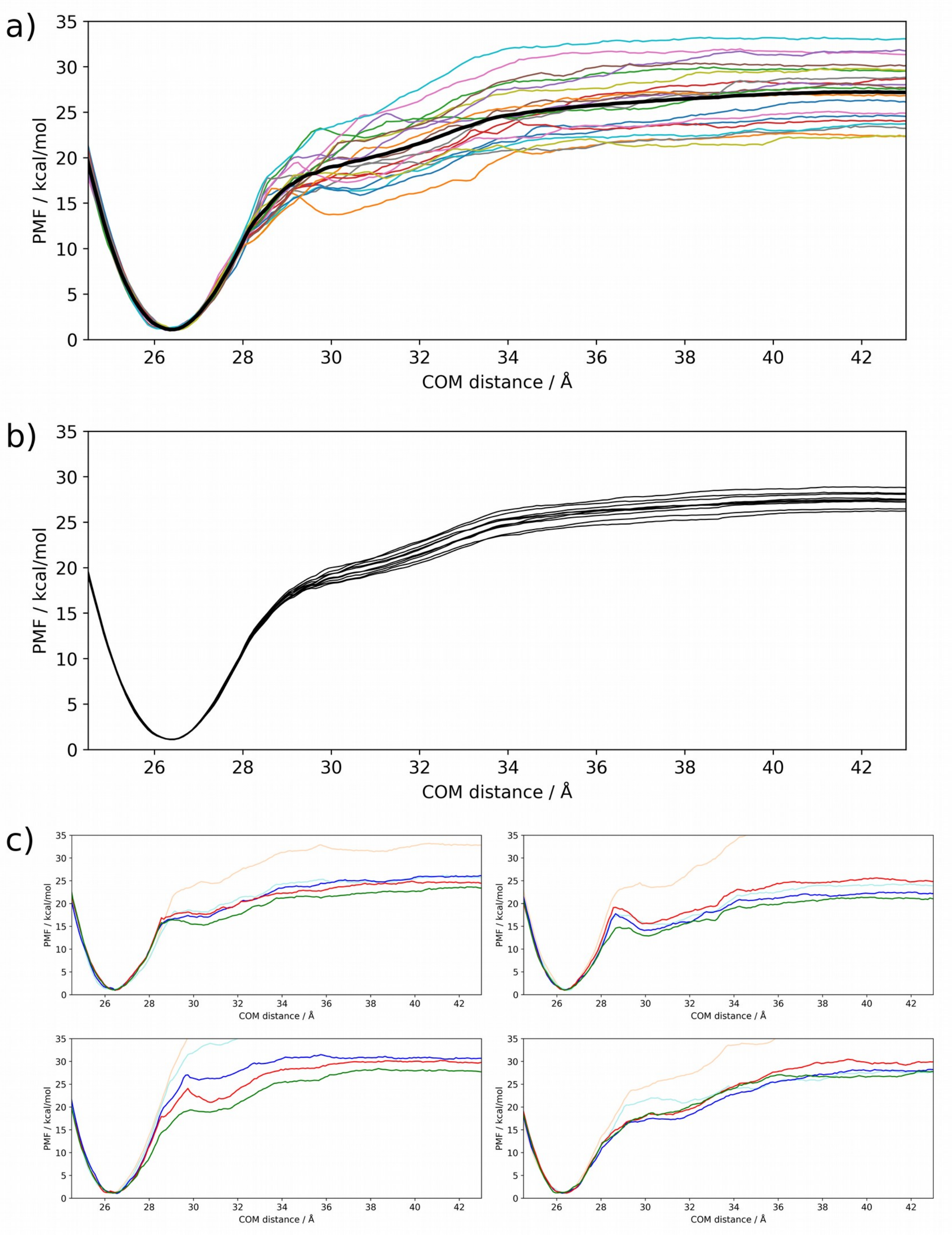

Figure S4: PMF curves of the US runs. a) The PMF curves vary for the 20 runs (colored lines). The PMF calculated from the combined runs is shown as black line. b) 10 bootstrapping resamples of the US runs have been produced by randomly combining the different US runs. c) To assess the convergence of the US runs, the US windows are splitted into 10 ns segments and the PMF is calculated for these segments separately. The pastel colors (pink, light blue) represent the segments of 0–10 ns and 10–20 ns respectively, which have been removed for the clustering and analysis of the simulations. The blue (20–30 ns), red (30–40 ns) and green lines (40–50 ns) show the PMF for the other segments. The result is shown for four runs.

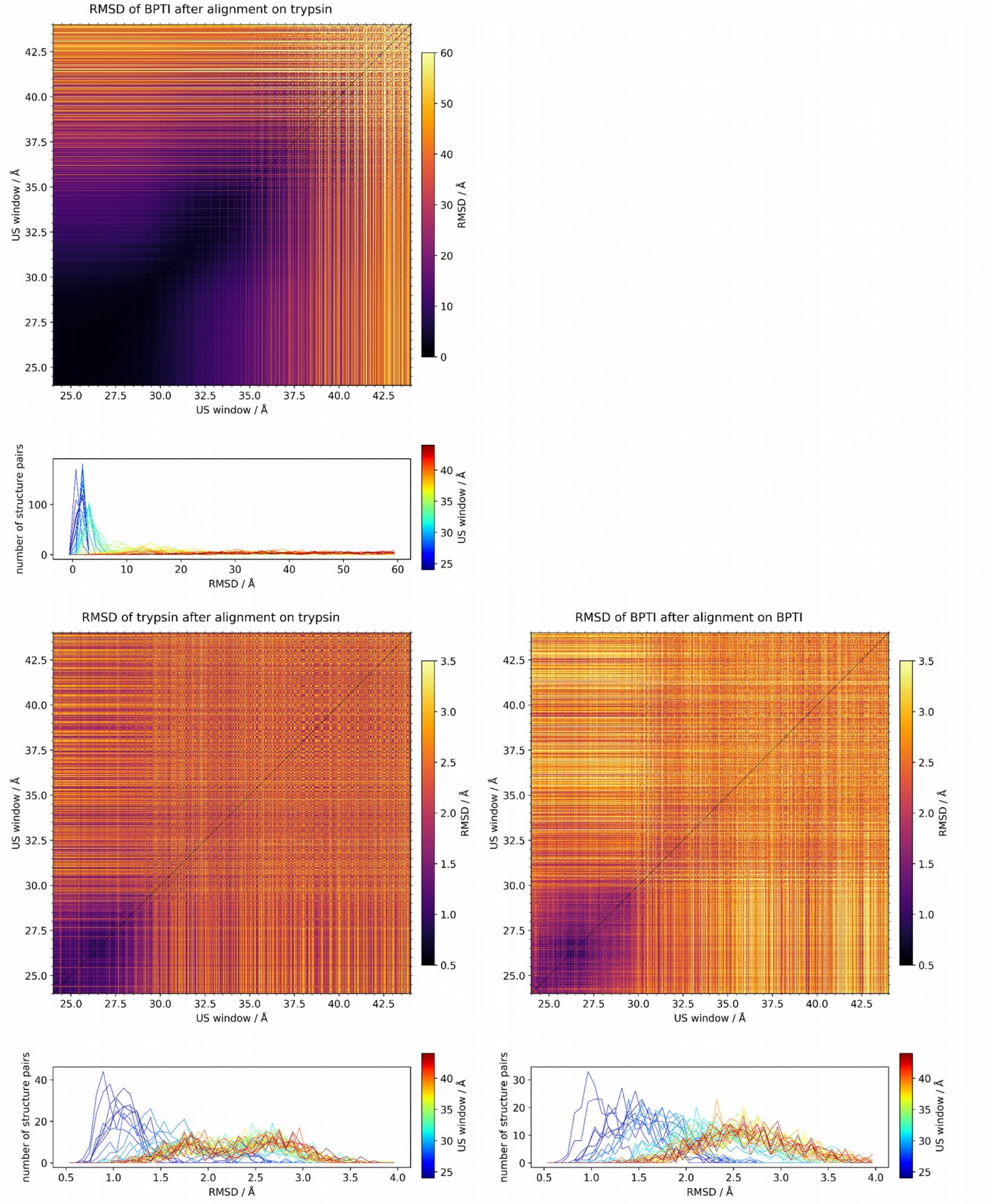

Figure S5: RMSD between the starting structures of the windows of all 20 US runs. The RMSD is calculated of BPTI after alignment on trypsin (top), of trypsin after alignment on trypsin (bottom left) and of BPTI after alignment on BPTI (bottom right). 2D-RMSD plots show the RMSD between all starting structures, sorted by the COM distance of the US window. Additionally, the distributions of RMSD values between starting structures belonging to the single COM distances are shown. On the one hand, the RMSD plots reveal the variety of BPTI positions. As unbinding takes place, an increasing number of different BPTI positions appear in the starting structures. At the same time, the internal conformations do not vary strongly, as internal degrees of freedom are not boosted by the US. However, it is clearly visible that the starting structures at small COM distances are very similar to each other and more varying at the US windows with larger distances.

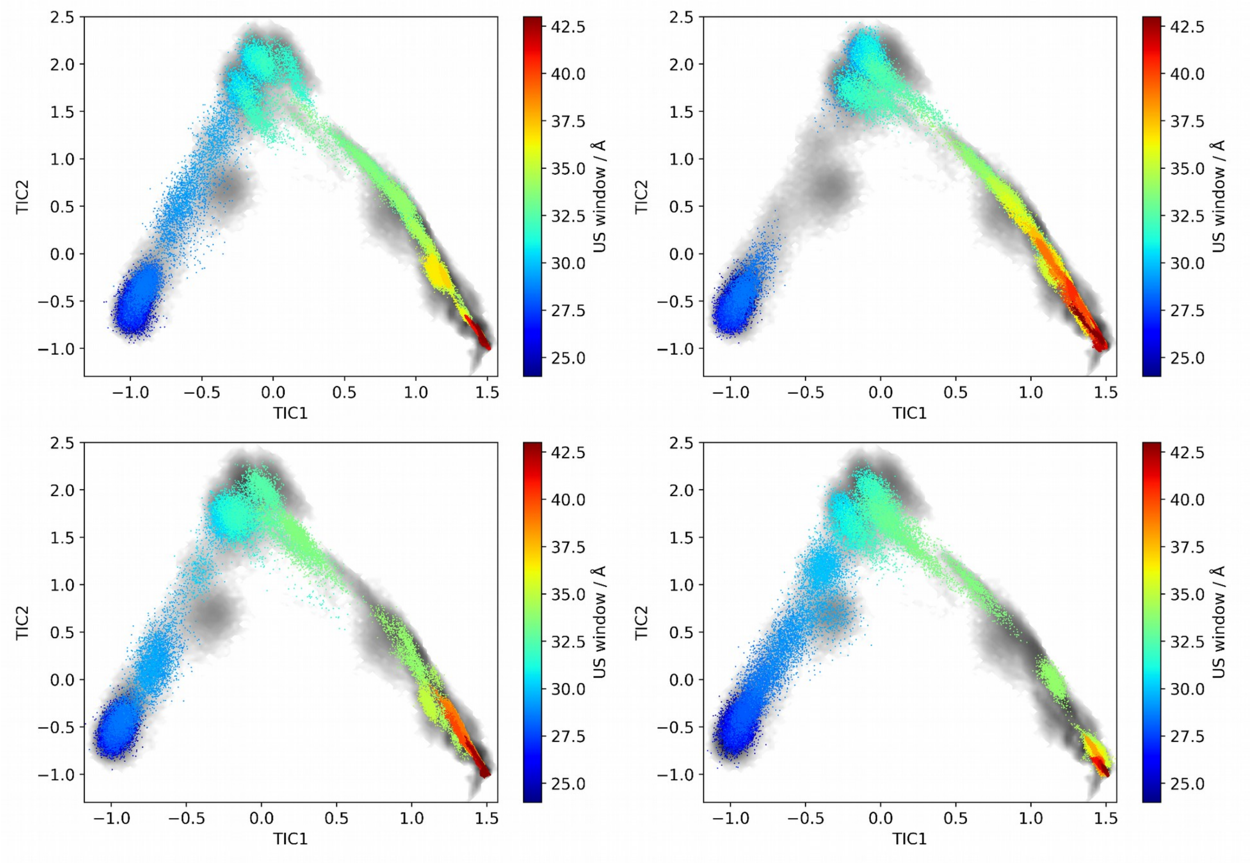

Figure S6: Projection of US runs into the TICA space. Roughly the same conformational space is occupied by the US runs (colored) as by the cMD simulations (grey) on which the TICA is based. Not all local minima are covered by all runs, suggesting that the sampled pathways vary. The result for four of the twenty runs is shown.

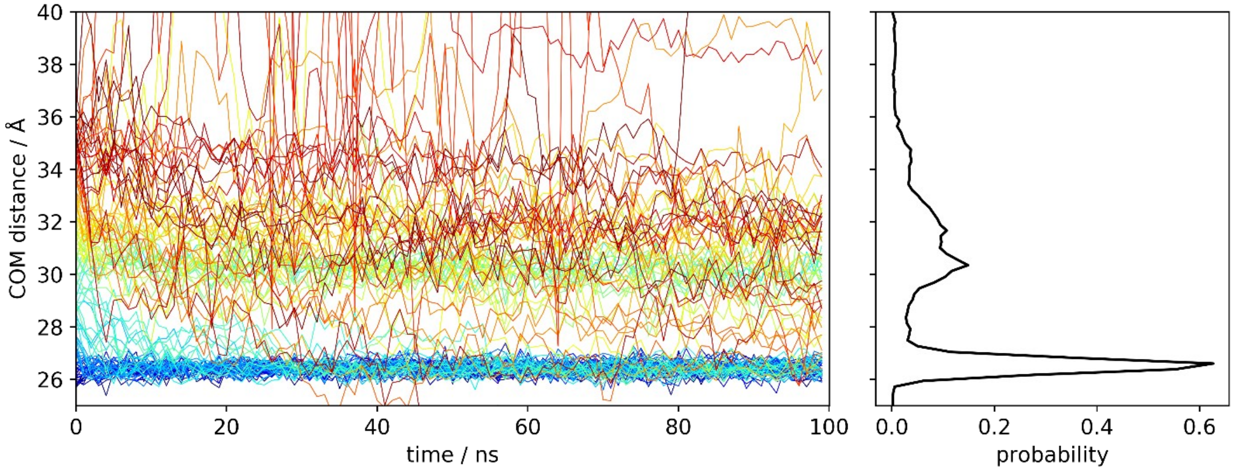

Figure S7: The progress of the cMD simulations started from conformations with different BPTI positions. The colors of the lines indicate from which umbrella window the starting structures originate, from dark blue being the starting structures from the smallest used CV window (25.0 Å) to dark red from the largest used CV window (36.0 Å). The panel on the right side shows the distribution of COM distances in these simulations.

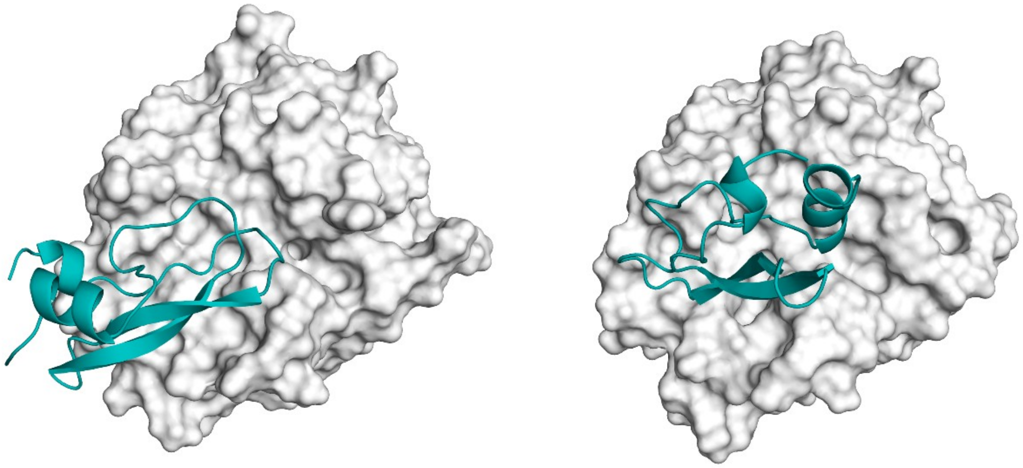

Figure S8: Representative structures, where BPTI has a flat orientation on the surface of trypsin. They lead to small (i.e., smaller than in the X-ray) COM distances. The structures were generated by clustering BPTI positions (alignment on trypsin), where the COM distance is smaller than 25.0 Å.

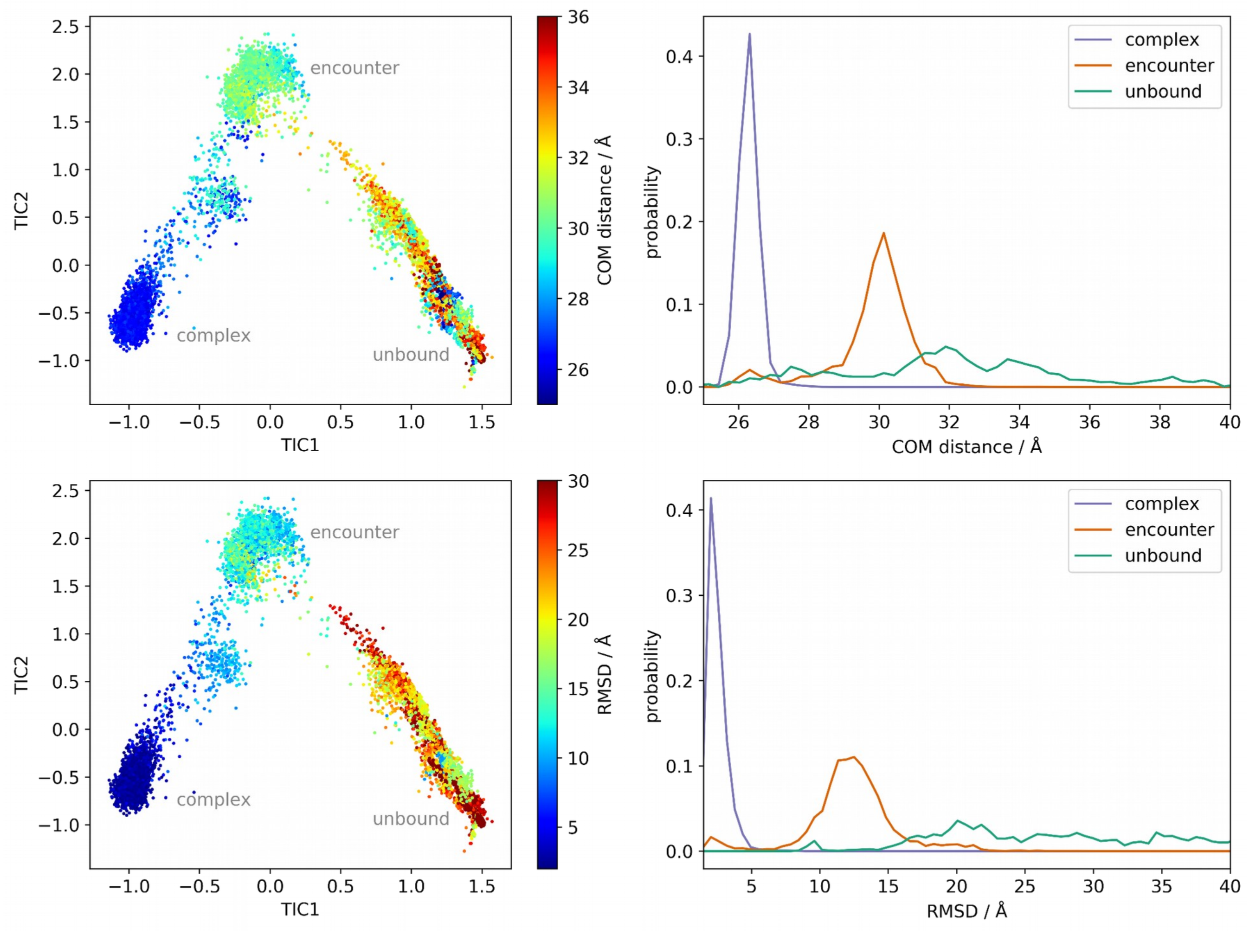

Figure S9: Distance of BPTI in cMD simulations. Left. The frames are projected onto the TICA space and colored according to the BPTI distance. Right: The distribution within the complex, encounter and unbound state is shown, as calculated from the MSM. Top: The distance between the centers of mass of trypsin and BPTI does not determine the state classification, e.g., in the unbound state very short distances can occur as BPTI can loosely associate to areas remote from the native binding site. Bottom: The RMSD values of BPTI (alignment on trypsin) in respect to its X-ray pose show that in the ensemble of the native complex, all configureations are similar to the X-ray structure, while in the unbound state, the vast majority of the positions of BPTI differ strongly from the native position.

TIC1

Ranking	Trypsin residue	BPTI residue	Maximal correla-tion of atom pair
1	W215	C14(P2)	-0.972
2	Q192	A16(P1’)	-0.970
3	Q192	K15(P1)	-0.969
4	C191	K15(P1)	-0.967
5	S214	C14(P2)	-0.965
6	S214	K15(P1)	-0.965
7	G193	A16(P1’)	-0.964
8	S190	K15(P1)	-0.963
9	D194	K15(P1)	-0.960
10	V213	K15(P1)	-0.959
11	S195	K15(P1)	-0.958
12	G193	K15(P1)	-0.957

TIC2

Ranking	Trypsin residue	BPTI residue	Maximal correla-tion of atom pair
1	G219	K15(P1)	0.559
2	C220	K15(P1)	0.501
3	F41	R17(P2’)	-0.406
4	H40	R17(P2’)	-0.386
5	C191	K15(P1)	0.380
6	G193	R17(P2’)	-0.372
7	W215	P13(P3)	0.370
8	Q192	T11(P5)	-0.370
9	Y39	I19(P4’)	-0.368
10	Y39	R17(P2’)	-0.361
11	F41	I19(P4’)	-0.359
12	S195	K15(P1)	-0.347

TIC3

Ranking	Trypsin residue	BPTI residue	Maximal correla-tion of atom pair
1	W215	P13(P3)	0.347
2	L99	P13(P3)	0.330
3	G216	P13(P3)	0.285
4	W215	K15(P1)	0.251
5	L99	C14(P2)	0.230
6	Q192	K15(P1)	0.203
7	S217	P13(P3)	0.197
8	L99	C38	0.193
9	Q192	G12(P4)	0.190
10	N97	R39	0.187
11	T98	R39	0.185
12	L99	R39	0.184

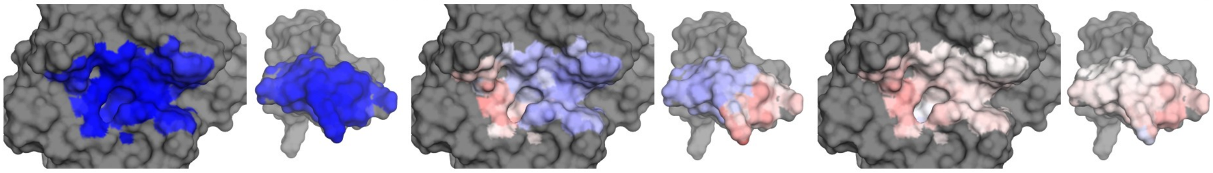

Figure S10: Correlation between the inverse distances of native atom pairs and the TICs. The table lists the largest correlations (according to absolute values) for TIC1, TIC2 and TIC3. It takes the largest correlation for each residue pair and neglects all following atom pairs of the same residues. (Clearly, neighboring atoms often show similar correlations, however to list them does not bear much additional information.) Underneath, each residue is colored according to the largest correlation, with which it correlates with the TIC, from -1 (blue), over 0 (white), to 1 (red).

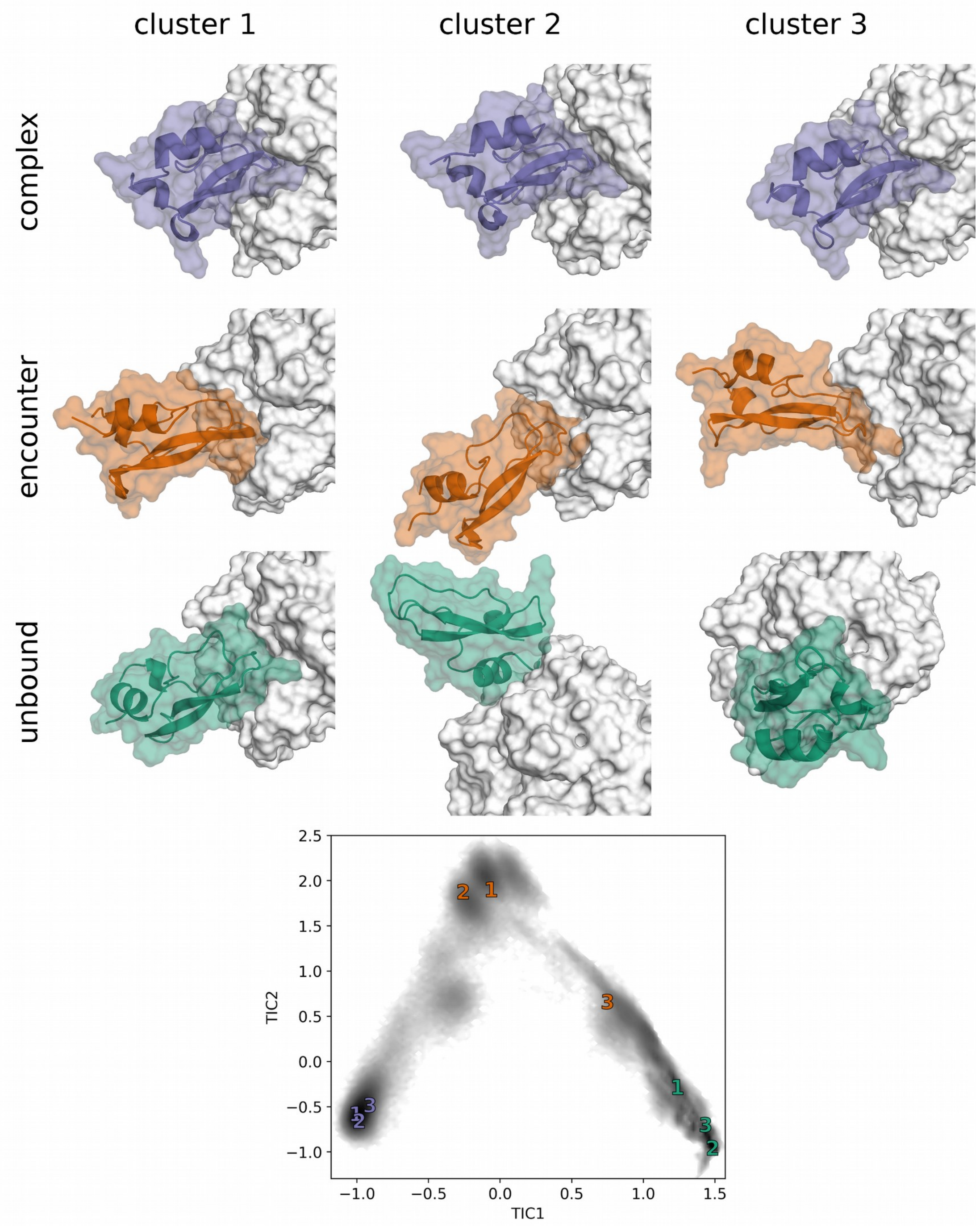

Figure S11: Representative structures of the complex (violet), encounter (orange) and unbound (green) state. Representative BPTI positions (RMSD of BPTI as cluster distance after alignment on trypsin) of the three most populated clusters are shown. The positions of these conformation in the TICA space are marked with the cluster numbers in the bottom panel.

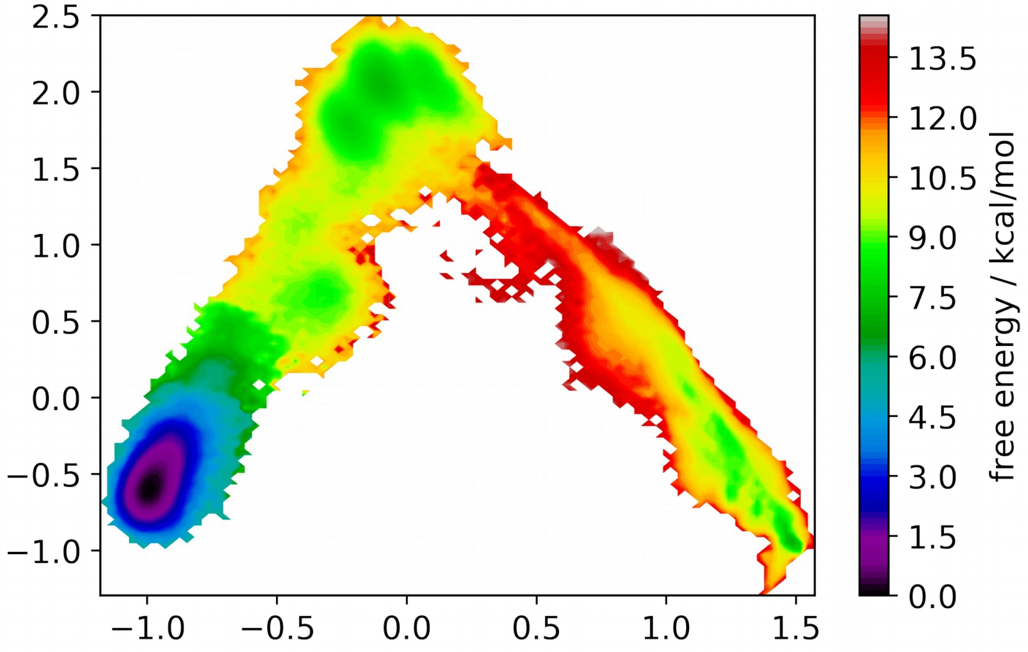

Figure S12: Reweighted free energy from MSM. The probability of the microstates calculated from the MSM has been used to reweight the distribution of the frames in the TICA space, resulting in a projection of the free energy.

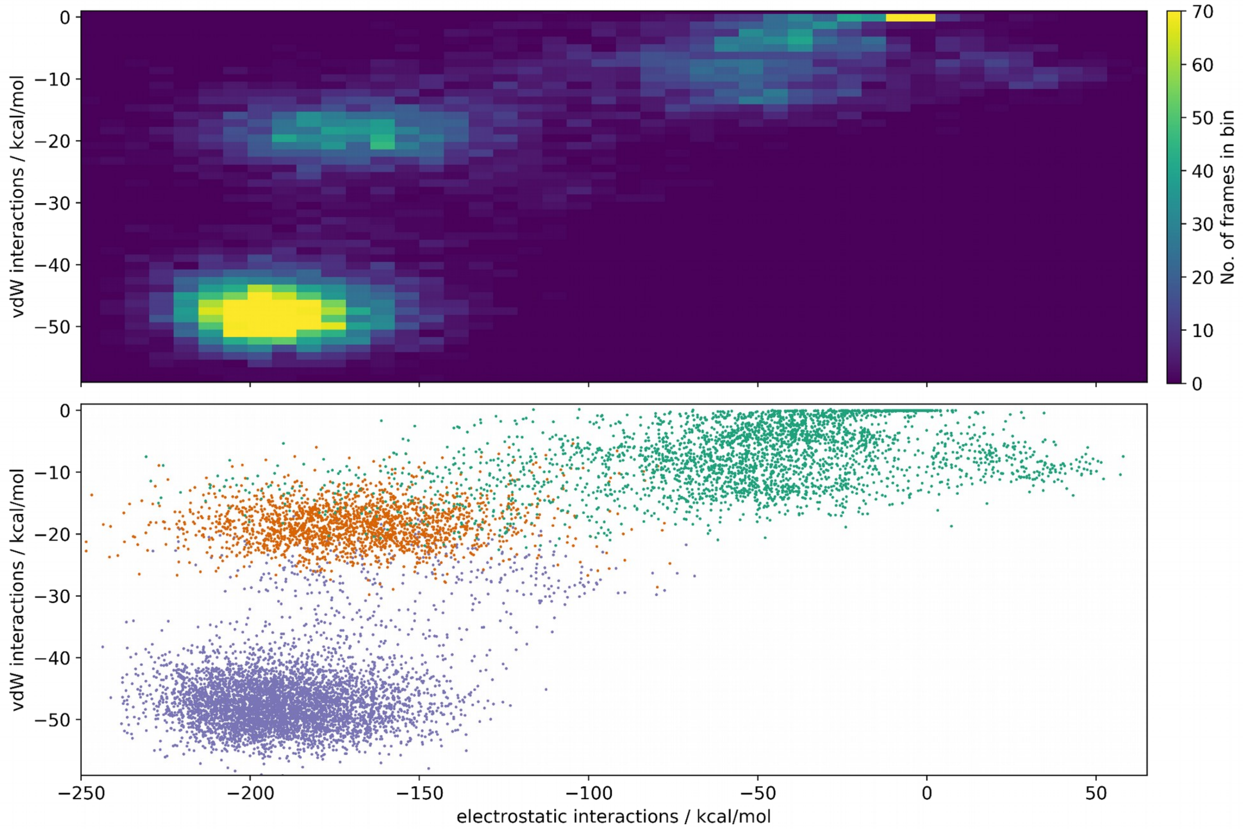

Figure S13: Van-der-Waals interactions and electrostatic interactions between the binding interface of BPTI and trypsin. Top: 2D-histogram of electrostatic and van-der-Waals interactions. Three major maxima in density can made out. Bottom: The position of the frames in the interaction space shows that the interactions mirror in the categorization into complex (violet), encounter (orange) and unbound (green) states.

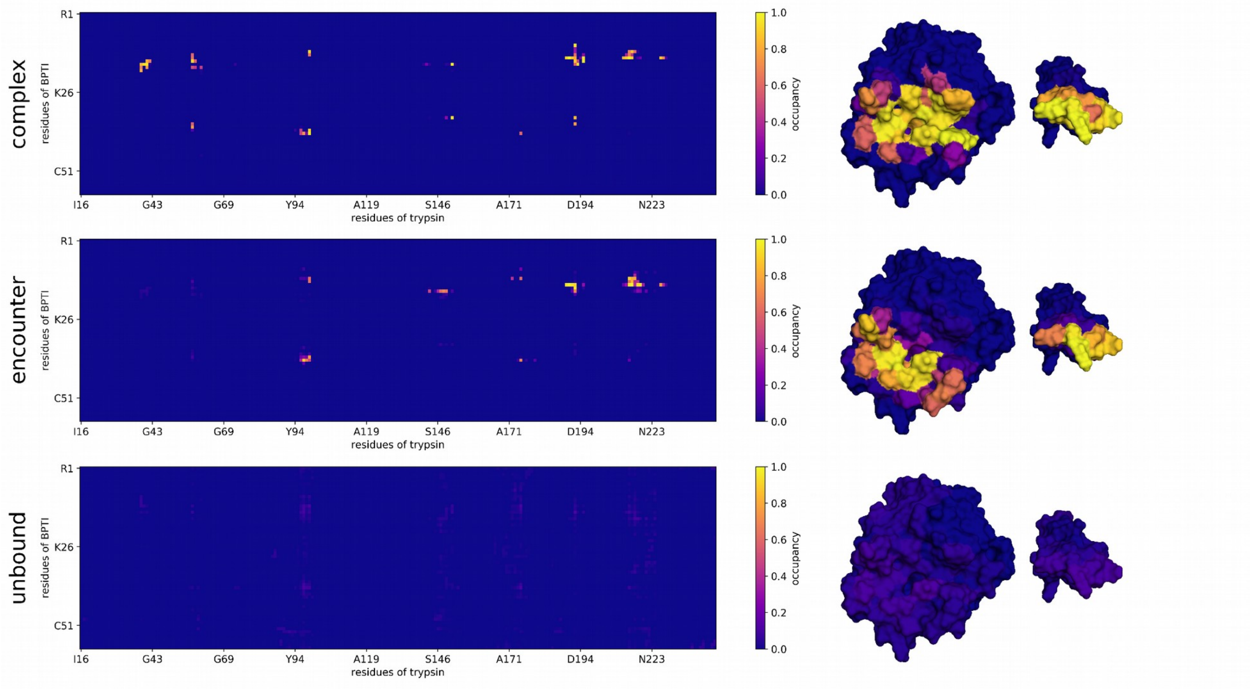

Figure S14: Contacts between trypsin and BPTI. The weighting of the contacts according to the MSM results in the division into the complex (top), encounter (middle) and unbound (bottom) state. Right: Coloring of the protein residue according to most stable contact, in which they are involved.

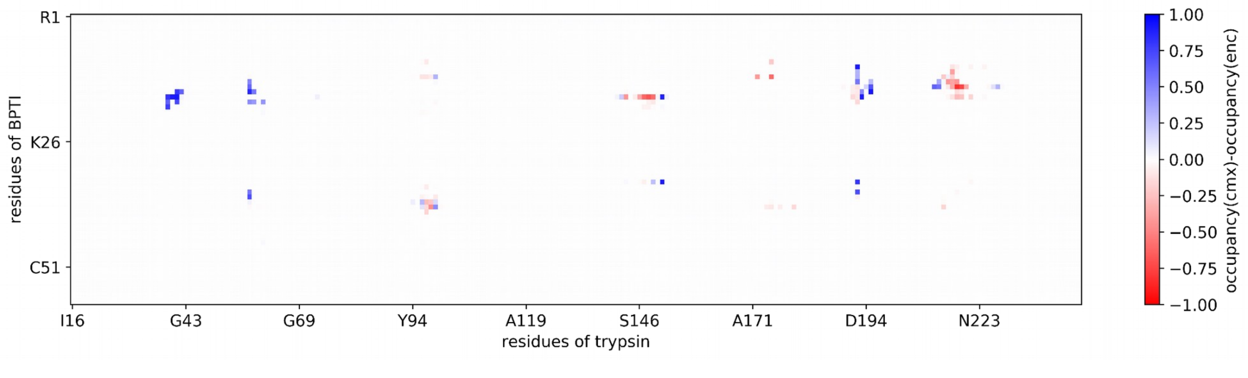

Figure S15: Difference in the residue-wise contacts between the complex and the encounter state. Blue coloring denotes contacts that have a higher occupancy in the complex state, red coloring residues that have a higher occupancy in the encounter state.

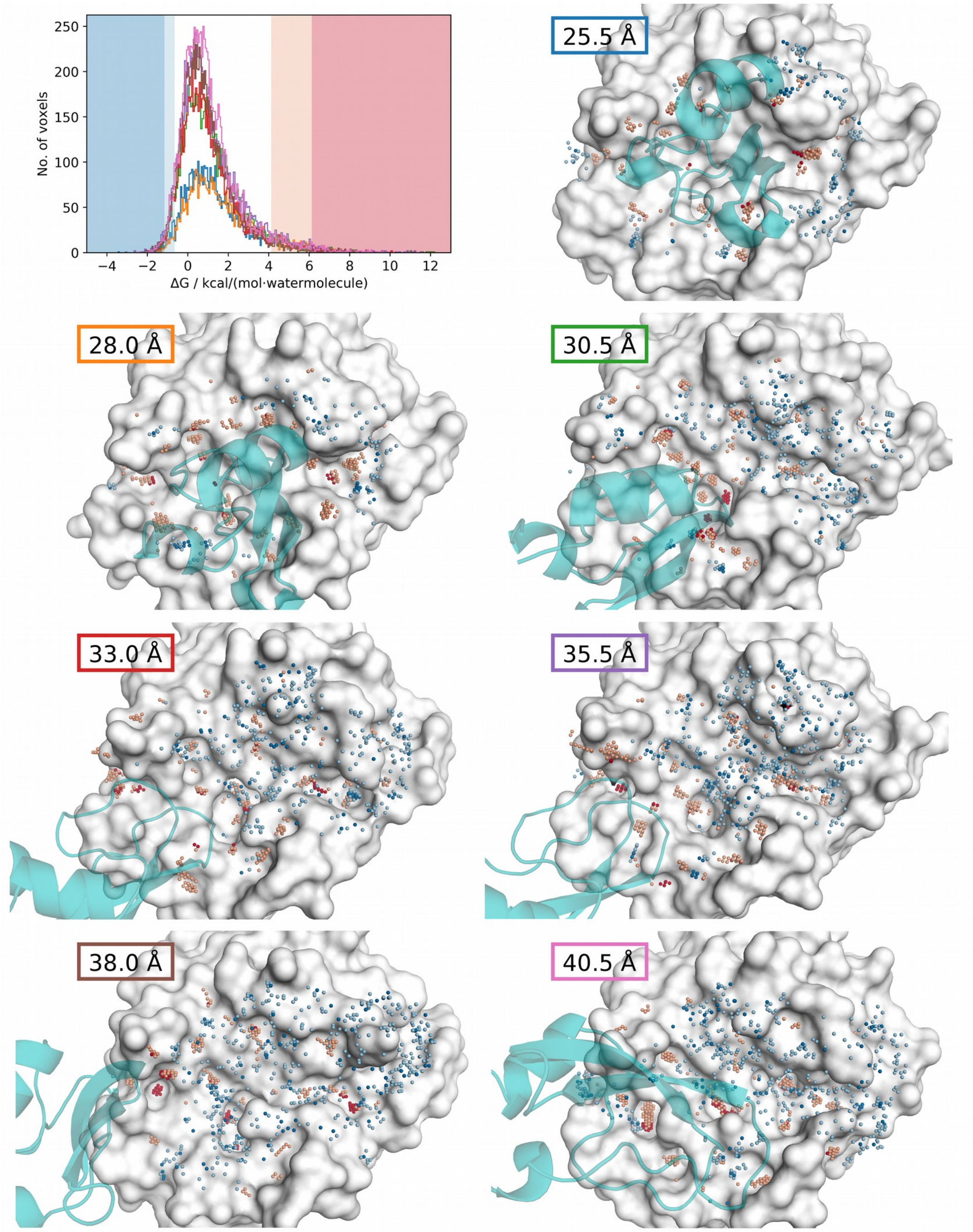

Figure S16: Free energy contributions of water molecules at the binding interface. Free energy contributions at grid points near the binding interface of trypsin have been calculated with GIST as described in the Methods section of the main document. The top-left panel shows a histogram of the free energy of water molecules at voxels with a significant water density (greater or equal to that of the bulk) at the different COM distances. The indicated 1st (blue), 5th (light blue), 95th (orange) and 99th (red) percentiles are used to restrict the shown voxel positions. The voxel positions are shown on the structures used for GIST and colored according to the free energy (red, orange, high free energy; blue, light-blue: low free energy).

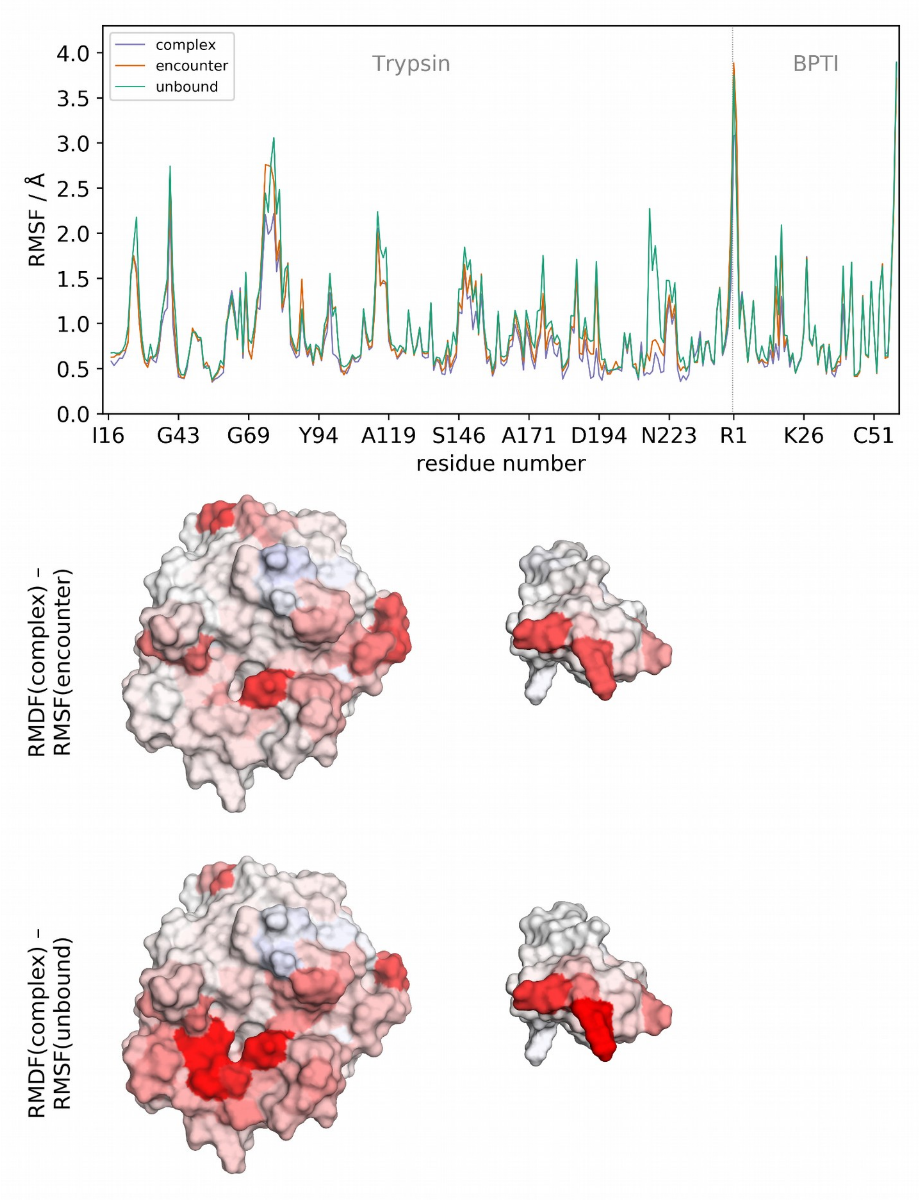

Figure S17: Residue-wise RMDF values within complex, encounter and unbound state. Top: RMSF values for trypsin and BPTI, middle: difference between the residue-wise RMDF values between complex and encounter, bottom: difference between complex and unbound from blue (-1 Å) to red (+1 Å). The calculation of the RMSF is calculated from 10,000 frames for each metastable state sampled based on the MSM.

Table S1: The 20 most highly populated contacts in the complex, encounter and unbound state. The frames have been weighted with the state probabilities from the MSM for the calculation of the occupancies.

Complex			Encounter			Unbound		
Trypsin	BPTI	occ.	Trypsin	BPTI	occ.	Trypsin	BPTI	occ.
Y151	R17	0.996	S190	K15	0.989	Q192	K15	0.123
S190	K15	0.994	G216	K15	0.987	Q175	R39	0.116
S195	K15	0.983	W215	K15	0.962	N97	R39	0.111
W215	K15	0.971	Q192	A16	0.953	Q175	P13	0.110
S195	A16	0.969	C220	K15	0.948	L99	P13	0.108
L99	R39	0.968	D189	K15	0.946	N97	K15	0.103
F41	R17	0.957	C191	K15	0.946	G219	R17	0.100
Q192	T11	0.953	G219	K15	0.934	S217	R17	0.100
G193	R17	0.949	Q192	K15	0.923	N97	C14	0.099
H40	R17	0.940	W215	P13	0.912	N97	C38	0.095
Y151	V34	0.938	N97	R39	0.892	N97	P13	0.091
H57	A16	0.929	G216	C14	0.832	W215	K15	0.086
L99	P13	0.925	S217	P13	0.812	T98	R39	0.085
D189	K15	0.910	G216	P13	0.806	L99	R39	0.084
L99	C38	0.909	L99	C38	0.765	Q175	C14	0.082
G216	K15	0.900	G226	K15	0.756	S96	K15	0.076
S214	K15	0.888	Q175	R39	0.697	Q221	R17	0.073
C191	K15	0.879	G148	R17	0.694	P92	R53	0.073
G226	K15	0.876	Q192	R17	0.686	H57	K15	0.072
Q192	A16	0.874	T98	R39	0.668	Y151	K15	0.071

Movie S1: Unbinding as sampled by an US run. BPTI is colored according to the RMSD to its native position, with green signifying a small RMSD and red a large RMSD. To obtain a continuous trajectory, only the first 10 ns of each window were used to make the video (which are not yet converged and not included in other analysis).

Movie S2: Example for a binding and unbinding event. Based on the MSM, a binding event (start at the unbound state and reaching the complex state) and an unbinding event (start at the complex state and reaching the unbound state) have been resampled. BPTI is colored according to the most probable metastable state to which the structure belongs to. The projection on the TICA space helps to understand the processes and to interpret the TICs.
