# Supplementary material for: Protein-Protein Binding as a Two-Step Mechanism: Preselection of Encounter Poses during the Binding of BPTI and Trypsin
Source: Biophys J. 2020 Jul 10;119(3):652–66. doi: 10.1016/j.bpj.2020.06.032 (PMC7399559; doi:10.1016/j.bpj.2020.06.032)
Supplement: Document S2. Article plus Supporting Material [file mmc4.pdf]

Biophysical Journal
Article

Biophysical Society

Protein-Protein Binding as a Two-Step Mechanism: Preselection of Encounter Poses during the Binding of BPTI and Trypsin

Ursula Kahler,[1] Anna S. Kamenik,[1] Franz Waibl,[1] Johannes Kraml,[1] and Klaus R. Liedl[1,*]
[1]Institute of General, Inorganic and Theoretical Chemistry, University of Innsbruck, Innsbruck, Austria

ABSTRACT Biomolecular recognition between proteins follows complex mechanisms, the understanding of which can substantially advance drug discovery efforts. Here, we track each step of the binding process in atomistic detail with molecular dynamics simulations using trypsin and its inhibitor bovine pancreatic trypsin inhibitor (BPTI) as a model system. We use umbrella sampling to cover a range of unbinding pathways. Starting from these simulations, we subsequently seed classical simulations at different stages of the process and combine them to a Markov state model. We clearly identify three kinetically separated states (an unbound state, an encounter state, and the final complex) and describe the mechanisms that dominate the binding process. From our model, we propose the following sequence of events. The initial formation of the encounter complex is driven by long-range interactions because opposite charges in trypsin and BPTI draw them together. The encounter complex features the prealigned binding partners with binding sites still partially surrounded by solvation shells. Further approaching leads to desolvation and increases the importance of van der Waals interactions. The native binding pose is adopted by maximizing short-range interactions. Thereby side-chain rearrangements ensure optimal shape complementarity. In particular, BPTI's P1 residue adapts to the S1 pocket and prime site residues reorient to optimize interactions. After the paradigm of conformation selection, binding-competent conformations of BPTI and trypsin are already present in the apo ensembles and their probabilities increase during this proposed two-step association process. This detailed characterization of the molecular forces driving the binding process includes numerous aspects that have been discussed as central to the binding of trypsin and BPTI and protein complex formation in general. In this study, we combine all these aspects into one comprehensive model of protein recognition. We thereby contribute to enhance our general understanding of this fundamental mechanism, which is particularly critical as the development of biopharmaceuticals continuously gains significance.

SIGNIFICANCE Protein-protein association determines the recognition of ligands, substrates, and inhibitors and thus controls manifold biological processes. The mechanisms involved in the binding processes are therefore of greatest interest and subject of numerous studies. Here, we detail the binding pathway of the protease trypsin and its inhibitor BPTI on atomistic level. We describe a two-step mechanism of binding, involving an intermediate encounter complex. In this encounter complex, the binding partners prealign their binding interface, which then enables further approaching. We describe the interactions and mechanisms that drive the initial association and the formation of the native complex. This study adapts a variety of fundamental concepts and combines them to form a comprehensive model for the binding partners trypsin and BPTI.

INTRODUCTION

The growing relevance of biopharmaceuticals (1) renders a comprehensive understanding of the fundamental mechanisms of protein-protein association, recognition, and binding of utmost importance. Initially, the recognition process between proteins was believed to resemble that between a lock and its key (2). Later theories acknowledge the significance of dynamics in the binding process. The induced fit model suggests that a reshaping of the active site takes place because of the interactions with the binding partner, leading to the formation of binding-competent conformations (3). Contrarily, the conformational selection theory claims that all protein conformations pre-exist within the dynamic apo ensemble, including the conformation of the bound state, although possibly only as a high-energy state. As the

Submitted March 6, 2020, and accepted for publication June 29, 2020.
*Correspondence: klaus.liedl@uibk.ac.at
Editor: Alan Grossfield.
https://doi.org/10.1016/j.bpj.2020.06.032
© 2020 Biophysical Society.
This is an open access article under the CC BY license (http://creativecommons.org/licenses/by/4.0/).

substrate recognizes and binds to this conformation, the removal of the stable complex from the apo equilibrium leads to a shift of the populations toward the binding-competent conformation (4–6). Motivated by cases in which an interplay of induced fit and conformational selection seems to guide protein binding (7), Csermely et al. (8) incorporate the induced fit mechanism into an extended conformational selection model, describing shifts of the energy landscapes as part of an adjustment process caused by mutual interactions (7–9).

The binding process takes place in different stages. In a first step diffusion leads to the association of the binding partners and formation of the metastable encounter complex (9,10), which is still mostly solvated (11). The association and alignment of the binding partners can be promoted by electrostatic steering, especially for charged proteins (11–18). In this context, Dagliyan et al. (18) investigate peptide-protein association with molecular dynamics (MD) simulations and find that omitting electrostatic interactions in most cases results in a decreased ratio between native-like encounter poses and transient encounter configurations. Electrostatic interactions shape a funnel-like energy landscape that directs the binding, pulling the interface together (19,20). Likely during this step, electrostatic interactions also contribute strongly to the discrimination between possible binding partners as described for the substrate recognition of serine proteases (20,21). After the formation of the encounter complex, a free energy barrier hinders a fast transition to the native complex (10,22). It is caused by the search for matching conformations and the desolvation of charged residues.

Using ensemble docking, Grünberg et al. (9) characterize the step after the association of the encounter complex as free conformer selection. Thereby, multiple different conformers can select their matching binding partners. This leads to a second intermediate form, which Grünberg et al. call the recognition complexes. They are similar to the native complex and feature a largely desolvated interface. In the last step, the near-native complexes can reorganize and refold to build the final, native complex (9,10). This process is characterized by a refinement of short-range van der Waals interactions, for which an optimal shape complementarity of the binding partners is essential. Hence, local side-chain dynamics play a decisive role in this final adjustment (18,23).

However, on detailed examination, a strictly stepwise description of the binding is likely an oversimplification of a far more convoluted process. The association can be described by a variety of possible binding pathways, consisting of weakly associated, transient encounter complexes (16,24,25), misbound configurations and a variety of intermediates (26,27).

Depending on the system, desolvation plays an important role in protein recognition processes (28). Desolvation of hydrophobic patches promote the binding, whereas desolvation of charged and polar residues slows it down (10). Camacho et al. scan rotational and translational space of ligands around receptors and evaluate the contributions of electrostatic interactions and of desolvation (12). They find that, especially for uncharged interaction partners, desolvation is a driving force in the binding process and can guide the formation of the complex. Thus, the differentiation between hydrophilic and hydrophobic surface regions can direct protein recognition and contribute to selectivity (29).

To investigate protein-protein association and recognition in atomistic detail, a number of different computational methods have been applied. Docking methods can be used to generate encounter complex poses (12,30–32). For example, Kozakov et al. (30) studied protein recognition via docking poses of a variety of systems. Interpreting them as intermediate states, they describe a reduction of accessible movement dimensions during association. Within the remaining dimensions, the encounter complexes are largely allowed free movement without high-energy barriers, which then facilitates the formation of the native complex.

Furthermore, Brownian dynamics simulations have been used to study association and encounters of proteins (13,33–35). A key advantage of this method is that the assumed approximations generally promote a highly efficient sampling. However, it oversimplifies or even neglects important effects of protein-protein interactions, like conformational dynamics and solvent effects, which most likely limits the achieved accuracy.

MD simulations provide a possibility to study protein-protein recognition in atomistic detail (36–40). However, for larger systems, the required timescale to observe the complete pathway between unbound proteins and complex conformations cannot be covered routinely because of the high number of degrees of freedom connected to the binding process (41,42). Enhanced sampling techniques that accelerate the sampling or restrict the sampled conformational space can provide the necessary speed up and a sound approximation of the underlying physics. Steered MD (43), restrained MD (26,44), and multiscale enhanced sampling (45) have been used to investigate association and dissociation of barnase and its inhibitor barstar, a well-studied model system of protein-protein recognition. For the same system, Plattner et al. (27) built a hidden Markov state model (MSM) of the complete branched pathway from association to native binding and calculated the kinetics of the respective transitions. Coarse-grained Monte Carlo simulations have been able to characterize transient encounter complex poses (46) that have been measured in paramagnetic relaxation enhancement studies (16,24). Here, we use umbrella sampling (US) (47) to overcome the sampling problem and to observe the dissociation of the serine protease trypsin and bovine pancreatic trypsin inhibitor (BPTI) (Fig. 1).

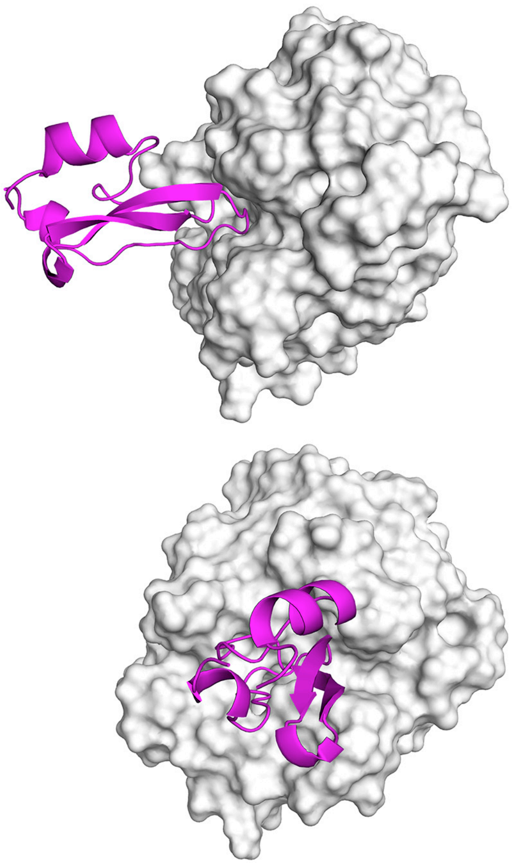

FIGURE 1 Complex between trypsin and BPTI. As depicted in the structure PDB: 3OTJ (66), trypsin (*white*), and BPTI (*magenta*) form a tight complex. Thereby, the substrate binding site of trypsin is occupied by BPTI, explaining the inhibitory effect. The top panel features a view from the prime site direction. The bottom panel depicts a frontal view, featuring a horizontal binding site of trypsin, extending from the nonprime site on the left to the prime site on the right. To see this figure in color, go online.

Trypsin is a serine protease of the chymotrypsin family and catalyzes the hydrolysis of consumed proteins as well as the activation of protease proenzymes as part of the digestive system (48). It is highly specific toward cleavage of peptide bonds after positively charged residues, i.e., lysine or arginine at the P1 position, but does not show strong substrate preferences at other subsites (49). Protease substrate amino acid positions are named with P1…Pn on the N-terminal side of the cleavage position and P1′…Pn′ on the C-terminal side. The binding subpockets of the protease are named accordingly Sn…S1|S1′…Sn′ (50). Computational studies of trypsin investigated the binding process of a small-molecule inhibitor and of the associated conformational changes (51,52). Here, we investigate the binding of trypsin to BPTI, a 58-residue Kunitz-type serine proteases inhibitor. Like trypsin, BPTI is a well-studied system. Its structure is one of the first resolved by x-ray crystallography (53) and it has been thoroughly studied with NMR experiments (54,55) and MD simulations (56). Association constants and binding free energies of the trypsin-BPTI complex have been measured experimentally (57–59) and estimated with MD-based methods (60,61).

As the subject of this article, we are investigating the formation of the complex between trypsin and BPTI. From US simulations we generate an ensemble of conformations that are highly diverse with respect to the relative orientation of BPTI and trypsin. We use structures along the dissociation pathway to seed unbiased simulations and build an MSM (27,51,52,62–65). It facilitates a kinetically grounded definition of the steps in the protein-protein recognition process from the prealignment of the encounter complex to the formation of the native complex. Along the different binding stages, we analyze conformational changes and protein-protein interactions, reporting on the driving forces and mechanisms of binding. We thereby provide a comprehensive and clear model of biomolecular recognition based on a well-studied model system.

MATERIALS AND METHODS

A schematic overview of the workflow of this study is depicted in Fig. S1.

Structure preparation

The trypsin-BPTI complex structure (Protein Data Bank, PDB: 3OTJ) (66) was used as basis for umbrella sampling simulations, the trypsin structure PDB: 3PTB (67), and the BPTI structure PDB: 5PTI (54) for the apo simulations. The structures were prepared with Molecular Operating Environment (MOE) (68), deleting cosolvents and ligands, keeping crystal waters, and adding missing hydrogen atoms with the Protonate3D tool (69). Solvent boxes of the TIP3P water model (70) were added with the LEaP application of AmberTools (71). A minimal wall distance of 12 Å was used for the apo simulations and one of 20 Å for the complex, ensuring sufficient space for the dissociation. The systems were minimized and equilibrated with Amber18 (71), following a thorough protocol previously developed in our group (72).

General simulation setup

All-atom MD simulations were performed with pmemd (73) in Amber18 (71) with the Amber14SB force field (74). The Particle Mesh Ewald (75) approach was used to calculate long-range interactions. The nonbonded cutoff was set to 10 Å. A uniform plasma was used to neutralize the charges. In the NpT ensemble, the temperature was kept constant at 300 K by a Langevin thermostat (76) with a collision frequency of 2 ps^{-1} and the pressure at 1 bar by a Berendsen barostat (77) with a relaxation time of 2 ps. The SHAKE algorithm (78) was used to restrain all bonds involving hydrogen atoms. The time step for the integration was set to 2 fs. Every 10,000th frame (every 20 ps) was saved for later analyses. For the apo simulations of trypsin and BPTI, 1 μs of production run was performed, respectively.

Implementation and analysis of US simulations

To separate BPTI from trypsin, US simulations starting from the equilibrated complex were performed. As collective variable (CV), the center of mass (COM) distance between the Cα-atoms of BPTI and of trypsin was used. The COM distance was chosen because it guarantees a minimum of local artifacts of the US sampling process, which are likely to occur for more locally defined CVs. Starting from the equilibrated structure, the umbrella windows extended between a COM distance of 24.0 and 43.5 Å with

a step size of 0.5 Å. The force constant of the harmonic spring potential was chosen to be 15.0 kcal/(mol·Å^2). Each window was run for 50 ns. After 10 ns of simulation time, the current conformation was extracted and used as starting structures for the next umbrella window. This procedure permits for an equilibration period at the previous distance and at the same time accelerates the sampling process by allowing partially parallel runs of the windows. Equilibration and US procedure were repeated 20 times to cover different possible pathways.

The WHAM (79) implementation of the PyEMMA (80) python package was used to reweight the US trajectories. The bin width was chosen to be 0.1 Å. The last 30 ns of each window were used for the analyses. The result for each of the 20 runs and the combined trajectories was visualized. To test the convergence of the single US runs, we performed trajectory splitting for all windows and calculated the potential of mean forces (PMFs) of all the segments. This allows an assessment on whether longer sampling of the US windows would lead to an improvement on the result. Additionally, we estimated the error of the 20 US runs by randomly combining the US runs to build bootstrapping resamples.

Seeding of cMDs

To allow an unbiased view at the mechanisms involved in protein-protein recognition and binding, the US runs were used to seed a large number of classical MD (cMD) simulations. Umbrella windows of the different runs that have the same target-value of the CV were combined and clustered to extract starting structures for the cMD simulations. The root mean-square deviations (RMSD) values of the BPTI atoms after alignment of the complex on trypsin were used as input for the clustering. With the hierarchical average-linkage clustering algorithm (81) of cpptraj (71) using a sieve of every 10th frame (for a faster processing), five clusters were generated for windows between 25.0 and 36.0 Å. The representative structures of the clusters, which consequently feature a large spread in BPTI orientations, were used as starting points for the unbiased simulations. From each of the 115 representative structures, cMD simulations of a length of 100 ns were produced. The Cα-COM regime over the course of these simulations and the distributions of the COM distances were visualized.

Analysis of cMD simulations and construction of a Markov state model

Time-lagged independent component analysis (82,83) (TICA) was performed with the PyEMMA package (80) using a lag time of 20 ns. As input features the inverse distances between native contacts (contacts between BPTI and trypsin atoms within 3.8 Å in the structure PDB: 3OTJ) were deployed. These features cover both, the large-scale unbinding movement and smaller side-chain rearrangements in the binding interface. The inverse distances thereby emphasize changes at small distances and filter out changes that take place at large distances (e.g., the movement of free BPTI). TICA finds the coordinates in which slow movements take place.

Based on the three time-lagged independent components (TICs) with the largest eigenvalues, we clustered the trajectories with the k-means clustering algorithm into 300 clusters to get discretized trajectories. They have been used to build a Bayesian MSM with a lag time of 20 ns. MSM construction and analysis have been performed with PyEMMA (80). The choice to use three time-independent coordinates is based on the distribution of values within the TICs (Fig. S2). The first three exhibit distinct maxima and minima suggesting high significance for the segregation of states that is less pronounced in the subsequent TICs. The number of clusters and the lag time were chosen based on implied lag time plots (Fig. S3; (84,85)). A lag time of 20 ns was chosen as the estimated slowest timescales are approximately independent of the lag time at that point. To simplify the MSM, PCCA++ (86) was performed, resulting in a three-state model. Although the slowest transition in the system is clearly that between the complex state (including the encounter state) and the free proteins, the gap between the second slowest and the third slowest timescale is still large, resulting in the three metastable states presented. With a Chapman-Kolmogorov test (84,87), the MSM has been evaluated (Fig. S3). Based on the MSM, the stationary probabilities and the dissociations constant have been estimated. With 10 bootstrapping samples (randomly combining the 115 cMD simulations), the confidence interval of the dissociation constant has been calculated.

For the visualization of BPTI positions, which have a small COM distance but are not typical native complex structures, we extracted frames with a COM distance lower than 25 Å and identified two representative structures with the hierarchical average-linkage algorithm of cpptraj (71). We used the RMSD of BPTI after alignment on the Cα-atoms of trypsin as clustering criterion.

To identify representative structures of the complex, encounter, and unbound state, we wrote out 10,000 frames according to the probability distribution of the microstates to be in each metastable state. These conformations were clustered structurally with the hierarchical average-linkage algorithm of cpptraj (71). Again, as input the RMSD values of BPTI, after alignment on trypsin were used. For the figure in the main text, one representative structure for each metastable state (complex, encounter, unbound) was extracted. For the supporting figure, to visualize the diversity of the states, 25 output clusters were generated and the representative structures of the three most populated clusters shown. All visualizations of structures were rendered with pymol (88).

The distribution of COM distances and RMSD values (again RMSD of BPTI after alignment on trypsin) in the TIC space and in the metastable states was determined to check the reasonability of the projection and state definition from the PCCA++ method. This state definition provides a fuzzy clustering and therefore probabilities of these states to be in each one of the microstates/k-means cluster and not a unique assignment. These probabilities were used to weigh the properties that have been determined by analysis of the trajectories when shown separately for the three states throughout the article. For visualization purposes, to avoid overcrowding in the TICA plot, cMD frames were extracted every 1 ns (in total 11,500 frames). Analogically the plots and distributions that characterize the binding process (described in the next paragraphs) have been prepared.

For quantitative analyses of the binding process, the electrostatic and van der Waals interaction were calculated with the lie command of cpptraj (71) with a distance cutoff value of 20 Å. To focus the analysis on the binding interface, only atoms that are within 3.8 Å of the other binding partner in the structure PDB: 3OTJ were included in the calculation. The results were projected on the TIC space. The probability distributions of the MSM has been used to weigh the average values and the standard deviations for the separated states. The ABPS plugin (89) of pymol (88) was used to visualize the electrostatics of the proteins. Similarly, the numbers of water molecules within the first (cutoff of 3.4 Å) and the second solvation shell (between 3.4 and 5.0 Å) of the binding interface (atoms within 3.8 Å of the other binding partner in the structure PDB: 3OTJ) were calculated. These values are the default settings for the calculation of watershells in cpptraj. The division between the solvent shells at 3.4 Å corresponds to a local minimum in the radial distribution function of water for the O-H distance, making it a reasonable value for this separation, also when looking at protein distances (including all atoms in the calculation). The angle between the binding sites was obtained by calculating the principal axes of the binding interface atoms with the "principal" command in cpptraj (71). The principal axes associated with the smallest eigenvalues point along the binding cleft of trypsin and the binding sequence of BPTI respectively. The angle between the vectors is small in the native complex as the binding partners are aligned. Histograms that show the distribution of angles within the metastable states according to the MSM have been plotted with a bin width of 1.8°.

Contact time series between the trypsin and BPTI residues were determined with cpptraj (71) with a distance cutoff of 3.8 Å. The contacts have been weighted with the probability distributions in the metastable MSM states and separate occupancies for the complex, encounter, and unbound states have been calculated. The occupancies and differences in

occupancies were visualized in contact maps and for an easier structural interpretation plotted on protein structures. The 20 most populated contacts for each cluster were listed in Table S1.

We clustered the cMD trajectories together with the apo simulations (frame offset of 1 ns for all simulations) with focus on structural differences within trypsin and BPTI. The apo simulation have been included as a control for the unbound state. For the purpose of this clustering that focuses on the internal conformational changes, we used the RMSD after alignment on the Cα-atoms of the respective protein. With the hierarchical average-linkage algorithm of cpptraj (71), 10 clusters were produced for each binding partner. For the complex, encounter, and unbound states, the occupancies were reweighted with the MSM. Representative structures of the three most populated clusters were displayed.

Residue-wise root mean-square fluctuation (RMSF) values were calculated based on the 10,000 frames that have been extracted for the complex, encounter, and unbound states as described above. The RMSF values have been determined after alignment on Cα-atoms of the respective protein (trypsin/BPTI). They consider the fluctuations of all atoms and characterize internal conformational flexibilities. The differences of the encounter and unbound clusters to the complex cluster were plotted on the structures of BPTI and trypsin to show differences in the conformational diversity of the states.

To extract solvent free energy data along the binding pathway, the grid inhomogeneous solvation theory (GIST) (29,90,91) was applied. For this purpose, we set up MD simulations with positional restraints of 1,000 kcal/(mol$\cdot$Å^2), starting at representative structures of the US (clustering of US as previously descripted, but with one single-output cluster) at seven different COM distances in an interval of 2.5 Å. After 50 ns of simulation time, we applied the GPU implementation of GIST (29) (GIGIST) in cpptraj (71) on 10,000 equally spaced frames (corresponding to a frame interval of 5 ps) with a grid spacing of 0.5 Å. For further analyses, we only considered voxels within 6.0 Å of binding interface atoms of trypsin. We limit the analysis to voxels with the same or higher water density as can be found in the bulk. According to the distribution of free energy values of the voxels, we visualize favorable (low free energy) and unfavorable (high free energy) water positions.

RESULTS

Sampling of the unbinding process

To investigate the factors involved in binding and unbinding processes, we chose the interaction partners trypsin and its inhibitor BPTI as model system. Starting at an x-ray structure that depicts the complex, US simulations were used to pull protease and inhibitor gradually apart. We used the distance between the COM of both proteins' Cα-atoms as a CV, ensuring a minimal disturbance of the binding interface itself. By performing 20 separate runs, different possible unbinding pathways were covered (compare to Figs. S4, S5, and S6). The runs are reweighted with the weighted histogram analysis method WHAM (79). The resulting PMF is shown in Fig. S4, together with an evaluation of simulation convergence and error estimation. Video S1 shows the unbinding process of one of the US runs.

To allow an unbiased view at the mechanisms involved in protein-protein recognition and binding, the US runs are used to seed a large number of cMD simulations, similar to other approaches used previously (65,92–94). Fig. S7 shows the COM distance in the unrestrained cMD simulations. All simulations that are started from complexes with small COM distances between trypsin and BPTI (COM distance $<$ 27 Å) stay stably bound during the 100 ns simulation time. Also, simulations with COM distances between 27 and 29 Å converge to smaller COM distances very fast and build a close complex. Simulations with larger starting COM distances (COM distance $>$ 30 Å) either converge at COM distances between 30 and 33 Å or change the COM distance without occupying any particularly stable position. In the latter case, BPTI is no longer strongly influenced by interactions with trypsin and can diffuse freely. Taken all simulations together, a COM distance of ~26.5 Å is clearly preferred. Although the simulations avoid COM distances of around 28 Å, a distance between 30 and 33 Å is again favored.

Once BPTI has left its binding position and can change its orientation freely, it can also adopt a flat position at the surface of trypsin (Fig. S8). This can lead to COM distances that are smaller than the distance in the native complex. Because the COM distance is not able to distinguish native binding poses and these transient configurations, it is unsuitable as the sole descriptor of the binding process.

Defining states via kinetics

To identify stable states along the binding process, we apply TICA (82,83) on the inverse distances of the native contacts. The inverse distances are well suited to identify small differences between conformations where trypsin and BPTI are close (i.e., the distances are small and the inverse distances are large), while not emphasizing differences in unbound conformations (where the inverse distances are small). TICA then retrieves the coordinates that contribute to the slowest changes in these original coordinates.

Fig. 2 shows several distinct density maxima in the TIC space. The highest maximum, on the left side of the TICA plot at TIC1 $= -1.0$ and TIC2 $= -0.6$, corresponds to bound conformations. For comparison, the projection of an equilibrated structure lies at TIC1 $= -0.94$ and TIC2 $= -0.36$. The density maximum at TIC1 $= 1.5$ and TIC2 $= -1.0$ (right side of the plot) contains conformations in which BPTI is distant from the native binding site. Moving from this unbound conformations toward the maximum at TIC1 $= -0.1$ and TIC2 $= 2.0$ corresponds to an alignment of BPTI with the binding site of trypsin. TIC1 correlates with the general progress of the binding process. Fig. S9 shows COM distances and RMSD projected on the TICA space. By comparison, TIC2 is rather associated with a rotating motion of BPTI, leading to correlations with different signs for the prime and the nonprime sites at the binding interface (Fig. S10).

We build an MSM (84,85) based on the first three TICs (Fig. S2) and use PCCA++ (86) to simplify the MSM to three metastable states along the binding pathway. They correspond to the observed major density maxima of the cMD simulations. We denote them as "complex," "encounter," and "unbound," supported by the

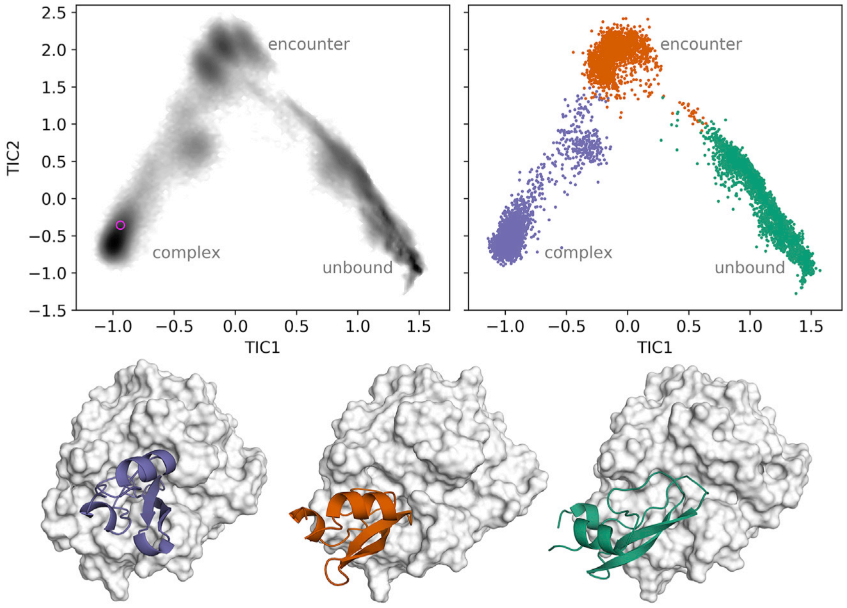

FIGURE 2 TICA of inverse native distances and state assignment by MSM. The upper left panel shows the distribution of the frames in the TIC space with the greyscale corresponding to their density. The magenta circle marks the projection of the x-ray structure after equilibration. The right panel displays an assignment of the simulation frames to the metastable state they have the largest likelihood to be part of (*violet*, complex; *orange*, encounter; *green*, unbound). Below, representative structures of these resulting states are shown. To see this figure in color, go online.

conformations that can be attributed to the states (Figs. 2 and S11). In the complex state, the orientations of BPTI are very similar to each other and also to experimentally determined complex. In the encounter complex, they are quite various and not so well aligned with the binding cavity of trypsin. Conformations in the unbound state are even more misaligned and there BPTI can also leave the binding site completely and diffuse into the solvent (Fig. S11).

The calculated stationary probabilities of the MSM states are 9.99984×10^{-1} for the complex, 1.2×10^{-5} for the encounter, and 3×10^{-6} for the unbound state. From these values, a dissociation constant of 2×10^{-14} M (lower and upper bounds of confidence interval estimated from bootstrapping: 1×10^{-17} M, 1×10^{-12} M, 95%) arises. Thereby, both the complex and the encounter states are considered to constitute the associated form as the slowest transition separates them from the dissociated, unbound state. The depth of the energy minimum of the complex is also clearly visible in the projection of the free energy surface calculated from the MSM and shown in Fig. S12. Video S2 displays an exemplary binding and unbinding event resampled with the MSM and tracks it in the TICA space.

Electrostatic and van der Waals interactions promote different binding steps

To investigate the driving forces in the binding process, we calculate the contributions of electrostatic interactions and van der Waals interactions between the binding interface of trypsin and BPTI (Fig. 3; for a two-dimensional histogram of the interactions see Fig. S13). For an equilibrated x-ray structure, the value of the electrostatic interactions is −212.8 kcal/mol and the value of the van der Waals interactions −48.8 kcal/mol.

The electrostatic interactions contribute significantly stronger to the binding than van der Waals interactions. This is expected considering that the binding interface of BPTI is lined with positively charged residues, whereas trypsin's binding cleft is charged negatively. Particularly noteworthy is the salt bridge between the P1 residue of BPTI, K15, and residue D189 in the S1 pocket of trypsin, which is present in the native complex.

In the unbound state, the electrostatic interactions between BPTI and trypsin are comparably weak. However, the values within the state vary a lot, as it includes configurations in which the proteins are relatively close as well as configurations in which they are far apart and rotated (Figs. 3 and S11). As the encounter complex forms, the electrostatic interactions strongly increase.

Van der Waals interactions at the protein interface play a minor role in the transition from the unbound state to the encounter complex. They significantly gain relevance as the encounter complex stabilizes to form the native complex.

Solvation of the binding interface continuously decreases during binding

The water molecules near the binding interface are counted to characterize the desolvation of the two binding partners (Fig. 4). Two different distance cutoff values are used to describe the first two solvation shells separately. For an equilibrated x-ray structure, the first solvation shell of the binding interface contains 67 water molecules and the second solvation shell 57 water molecules.

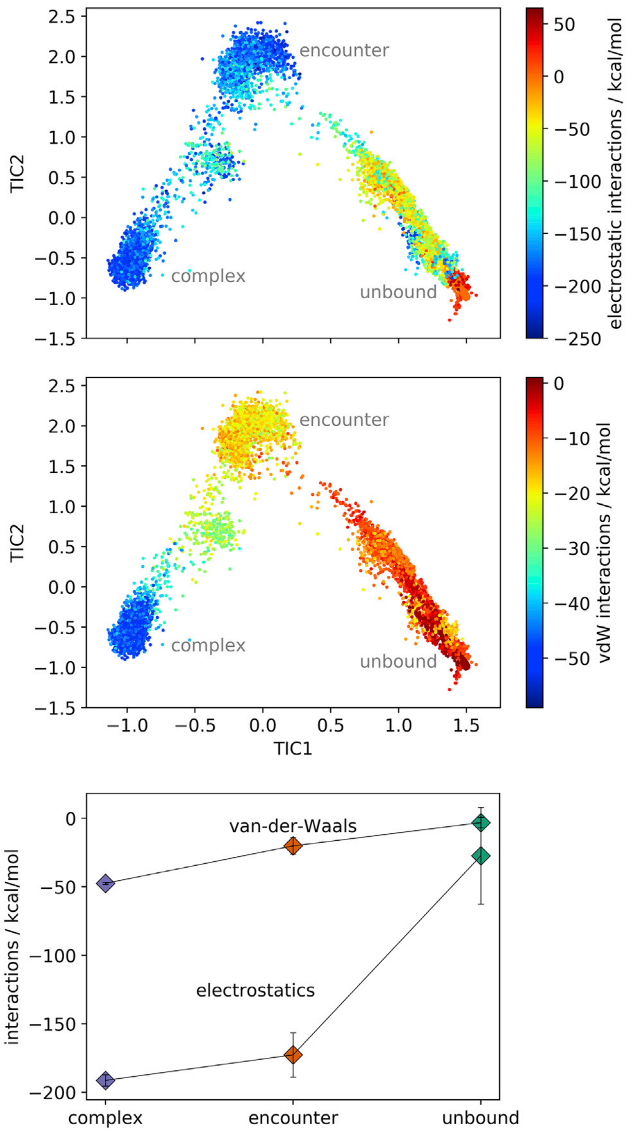

FIGURE 3 Electrostatic interactions and van der Waals interactions at the binding interface of trypsin and BPTI. Frames in TIC space are colored according to electrostatic interactions (*top*) and van der Waals interactions (*middle*). The bottom panel shows average and the standard deviations within the metastable states (complex, encounter, and unbound). To see this figure in color, go online.

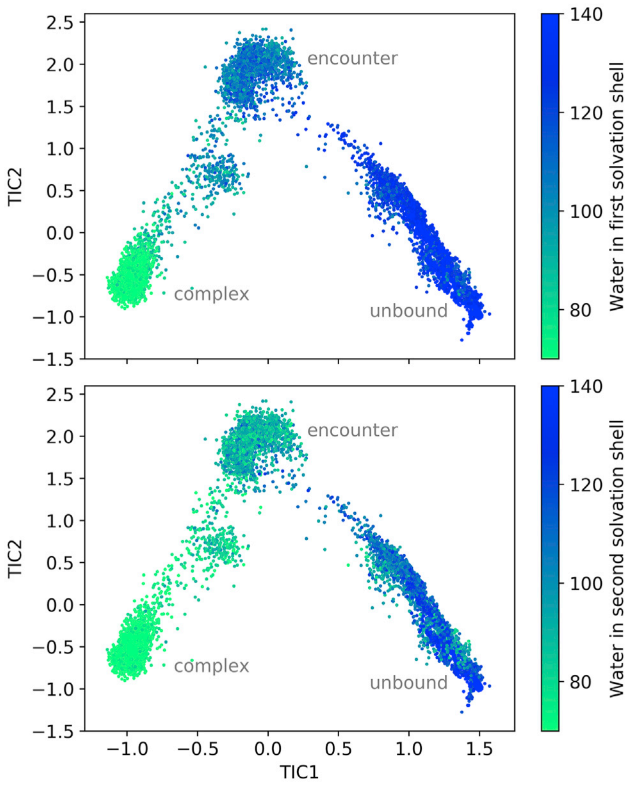

FIGURE 4 Solvation shells around the binding interface of trypsin and BPTI. The top panel shows the number of water molecules within a 3.4-Å distance to binding interface atoms (first solvation shell) and lower panel the number of water molecules between 3.4 and 5.0 Å from the binding interface (second solvation shell), depicted as colors of the frames in the TIC space. To see this figure in color, go online.

As the binding partners approach, water molecules are displaced from the interface between the proteins. The number of water molecules decreases rather uniformly during each binding step, i.e., in the transition between unbound and encounter states as well as in the transition between encounter and native complex. Arguably, in the step from unbound proteins to encounter complex, the second solvation shell is more impacted by the water displacement than the first solvation shell, whereas for the formation of the native complex, the first solvation shell has to be disbanded in the binding interface. In each of the clusters (complex, encounter, and to a lesser extent in the unbound state), the number of surrounding water molecules is relatively constant. This suggests that the states can be distinguished based on the solvation of the respective conformations and that the contributions of the solvation play an important role during the binding process.

Prealignment of the binding interface precedes complex formation

To enable binding, BPTI and trypsin must align so that their binding interfaces face toward each other. We determine the principal axis with the smallest eigenvalue of the binding interface atoms for both proteins and calculate the angle between these vectors to quantify the relative orientation of the proteins. The binding cleft of trypsin has the typical shape known from the chymotrypsin family, consisting of a series of pockets that recognize the peptidic substrates (95). BPTI, accordingly, binds to it with a sequence of residues. Consequently, both proteins' binding interfaces have a well-defined, elongated form. In the complex, this results in a nearly parallel orientation of their principal axes, associated with the smallest eigenvalues. Thus, the angle between the principal axes in the structure PDB: 3OTJ is 0.40°.

Fig. 5 shows that in the complex only a narrow range of angles close to 0° occurs. In the encounter complex, the range of angles is restricted as well, although not as strongly as in the native complex. There, the most probable angles are around 36°. The encounter complex rarely shows angles as are predominant in the native complex. In the unbound state, a wide range of angles is possible because trypsin and BPTI are almost randomly oriented.

Conformational changes during the binding process

To find whether conformational changes within the proteins occur during the binding process, we clustered the conformations of trypsin and BPTI separately based on their RMSD, together with simulations of the apo forms of trypsin and BPTI. The apo simulations are included as control, in which the possibilities of interaction between the proteins and of artifacts from starting the sampling at the complex structure are eliminated. The comparison with apo simulations also allow the interpretation of the result in terms of the dominant binding model, i.e., induced fit or conformational selection. At the same time, the classification into the complex, encounter, and unbound states enables a stepwise allocation of the events.

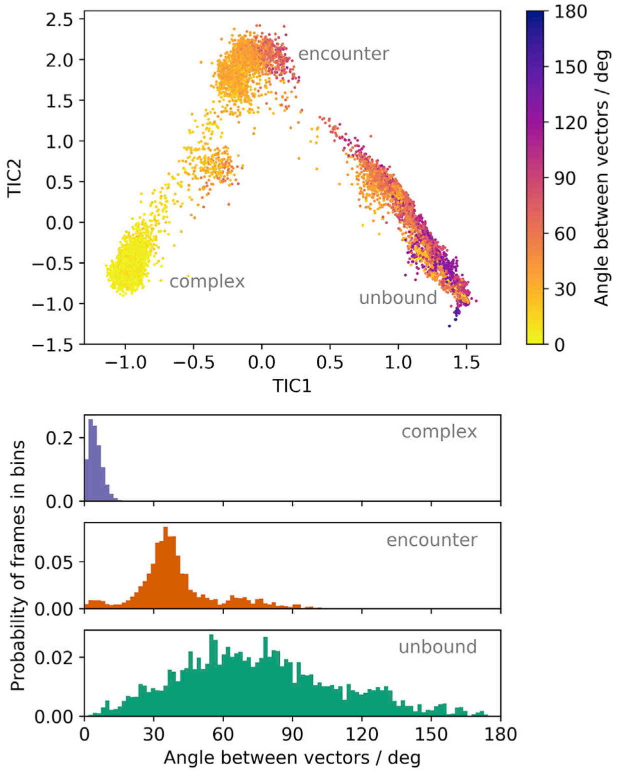

FIGURE 5 Angles between the binding cleft of trypsin and binding interface of BPTI. The angles plotted on the frames in the TIC space (*top*) show a prealignment of the binding interface, resulting in a fixed angle in the complex. This becomes particularly evident in the angle distribution within the metastable states, i.e., in the complex, encounter, and unbound states. To see this figure in color, go online.

Fig. 6 displays the result of the clustering for trypsin. The three largest clusters are present during the entire binding process. However, a detailed look at the distributions (Fig. 6 *b*) shows that the most populated cluster is accumulated in the native complex, while being present also, but to a lesser extent, in the encounter complex, in the unbound form, and in the apo simulation of trypsin. The second cluster (*yellow* in Fig. 6) is the most populated cluster in the unbound state and loses importance during the binding process, i.e., in the encounter complex and the native complex.

Structurally, the main difference between the clusters is observed for residue Y39, which builds the upper part of the prime site of trypsin's binding cleft. The position of its bulky side-chain dominates the clustering. In x-ray structures and the preferred conformation of the complex cluster, the side-chain is directed toward the S1' pocket. In other conformations, it points in the direction of more remote areas of the prime site or toward the solvent.

Fig. 7 shows the result of the clustering for BPTI. Only one cluster allows the formation of the complex between trypsin and BPTI. This conformation is also preferred for the formation of the encounter complex and present in the apo form.

The conformations of the residues K15 (P1) and R17 (P2′) show the largest structural variance. They are thus decisive for the clustering. Both side-chains are long and flexible and can take a variety of conformations. However, to bind to trypsin, both have to adopt a specific orientation so that they can fit into the S1 pocket and the prime site respectively. These conformations are already present in the apo form as part of a wider ensemble, but native binding is only possible when the side-chains match to trypsin conformations and do not cause clashes and, therefore, repulsion between the proteins.

DISCUSSION

As basis for nearly all physiological functions, proteins have to selectively recognize specific binding partners. Especially for drug design efforts, a thorough understanding of the mechanisms promoting protein-protein recognition is paramount, e.g., to create inhibitors and antibodies with high affinity, but also to hinder unwanted association causing agglomeration or severe side-effects. As a model system, we investigate the binding process between trypsin and its inhibitor BPTI and aim to understand the fundamental factors that contribute to recognition and binding in atomic detail.

With unbiased MD simulations, sampling of binding and unbinding could take hundreds of microseconds to seconds, which exceeds routinely viable simulation times by far. To enhance the efficiency of the sampling, we use US simulations to cover conformations along the path between complex and dissociated proteins and then construct an MSM

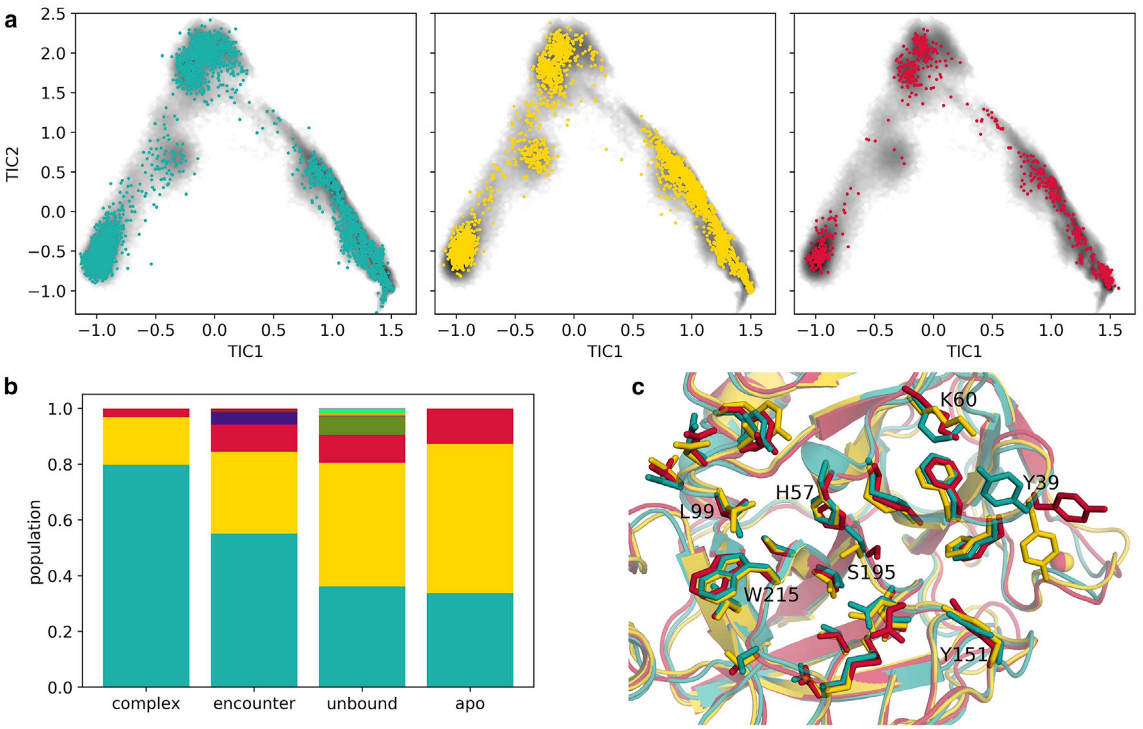

FIGURE 6 Conformational changes of trypsin during the binding process. (*a*) Membership of frames to the first- (*teal*), second- (*yellow*) and third-most-populated cluster (*red*). (*b*) Populations of the clusters (conformational clustering) within the complex, encounter, and unbound state, weighed with the state distributions from the MSM, and an apo simulation. (*c*) Representative structures of the clusters, with the side-chains of the residues that are part of the binding interface shown as sticks, highlight the structural differences. For better orientation, selected residues are labeled. To see this figure in color, go online.

based on these simulations. Similar approaches have been used previously (92–94), also with different enhanced sampling methods. Thereby, the choice of enhanced sampling method is rather incidental, as long as the extracted starting structures for the unbiased simulations are well distributed on the conformational space of interest and feature also high-energy structures near transition states allowing for transition between metastable states. Here, we chose US sampling for this purpose because it seems to be the obvious pick for the unbinding process, but it stands to reason that for example metadynamics simulations could have fared equally well. We perform 20 US runs to cover different possible association pathways. Figs. S4 and S6 show that the resulting pathways are indeed quite diverse, with different local minima and maxima occurring. The PMFs seldom follow the same regime. Trajectory splitting shows that after a certain equilibration period, the PMFs do not change strongly anymore (Fig. S4). Still, a convergence of the US simulation runs cannot be assumed within the 50 ns of simulation time per window and it is to assume that far longer simulations would lead to more equalized PMFs. However, comparing these results to the various US runs, which differ more strongly, we can assume that starting more runs is a more efficient way to cover possible transition pathways than extending window length in a single run. Poor convergence could also explain the lacking agreement between the free energy difference of unbinding calculated from the US (26.1 kcal/mol, confidence interval from bootstrapping: 25.2 kcal/mol, 27.7 kcal/mol, 95%), compared to the experimentally determined value (17.85 kcal/mol) (57), alongside with differences in experimental and calculation setup. Regardless of how sampling has been accomplished, the full convergence of the US is not essential for this work because the method is foremost used to seed cMD simulations and not to make quantitative statements. The regime of the PMF of many US runs follows the same as described by Hoefling and Gottschalk (26) for the unbinding of the barnase-barstar complex. They describe a steering region where the binding partners approach, followed by a local minimum, a transition state and finally the global minimum corresponding to the native complex. This trend is also mirrored in the distribution of the subsequently performed cMD simulations (Fig. S7). As the local minima and maxima occur in the US runs at different COM distances, they are less distinct in the combined PMF.

Although we simulate different configurations of BPTI and trypsin along the binding pathway, we do not capture very slow conformational changes within these proteins. For both, BPTI (56) and trypsin (52), long-timescale simulations have shown large conformational rearrangements, e.g., isomerization of disulfide bridges and large-loop

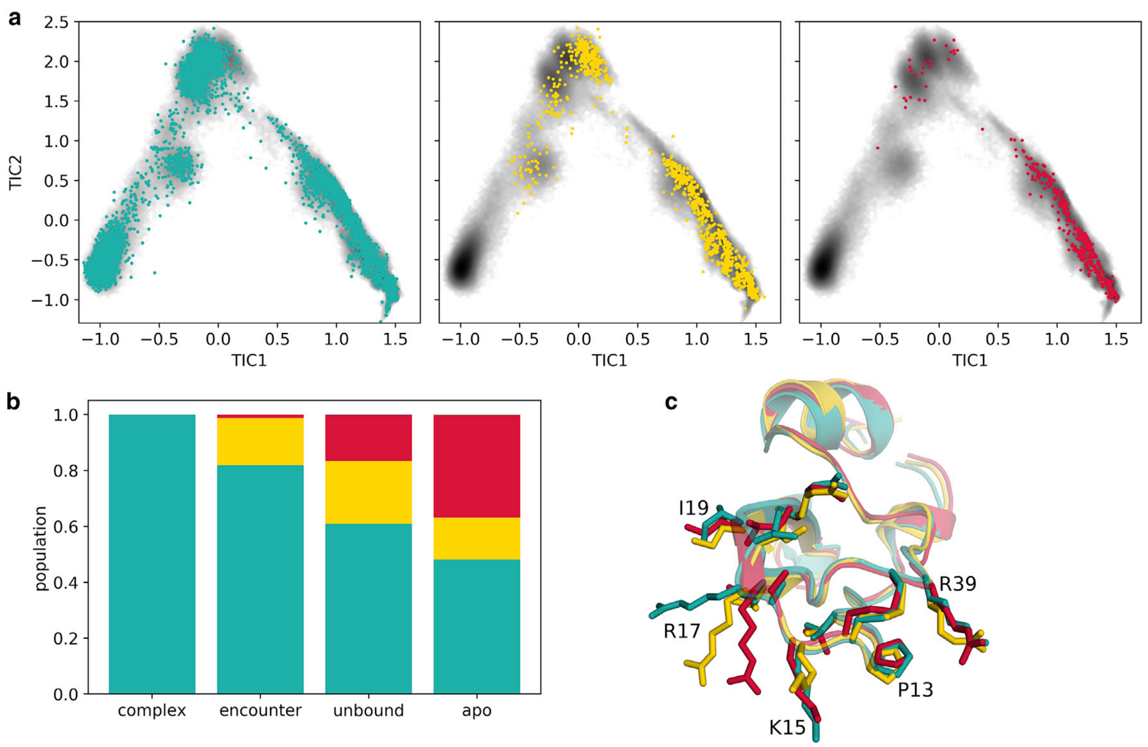

FIGURE 7 Conformational changes of BPTI during the binding process. (*a*) Membership of frames to the first- (*teal*), second- (*yellow*) and third-most-populated cluster (*red*). (*b*) Populations of the clusters (conformational clustering) within the complex, encounter, and unbound state, weighed with the state distributions from the MSM, and an apo simulation. (*c*) Representative structures of the clusters, with the side-chains of the residues that are part of the binding interface as sticks, show the structural differences. To see this figure in color, go online.

rearrangements. These motions can take hundreds of microseconds and are not covered by our simulations. The enhanced sampling technique and, in consequence, the cMD simulations focus on sampling different unbinding pathways and do not accelerate the conformational sampling within each binding partner.

We perform TICA on the cMD simulations to filter out fast transitions and focus only on the slow ones. An MSM provides a classification in states that is based on the kinetics of the system. The calculated dissociation constant, $K_d = 2 \times 10^{-14}$ M, is in good agreement with experimental measurements of $K_d = 6 \times 10^{-14}$ M(57) ($K_d = 5 \times 10^{-14}$ M(58)). However, the kinetics of the unbinding could not be reproduced, which is not overly surprising, considering that the half-life of the complex is ~8 months (58). Despite this issue, the slow eigenvectors of the transition matrix often remain meaningful despite errors in the estimation of the absolute timescales (96). Therefore, the classification into the metastable states, complex, encounter, and unbound is also kinetically grounded. In the literature, the definition of the encounter complex varies, but usually, it describes an intermediate along the binding pathway. We clearly find the presence of such an encounter complex ensemble within our state definition. We want to emphasize that rather than one clearly defined structure, the encounter complex is a diverse ensemble of conformations. This ensemble that we call encounter complex does not include transient encounter complexes, which are short lived and associate remote from the native binding site (16,24) but only prealigned conformations near to it. We do not discriminate between the transient encounter complexes and the unbound states but focus on later stages of the binding process, which are divided by major free energy barriers. We find that, by far, the slowest transition in the binding process is the association of the encounter, whereas the building of the native complex is faster.

The driving forces for the initial association and binding of trypsin and BPTI are clearly electrostatic interactions (Fig. 3). Especially in the association from the unbound proteins to the encounter complex, they strongly increase, corresponding to the frequently described electrostatic steering (12,14,15,26). The binding cleft of trypsin is negatively charged, and the binding interface of BPTI is positively charged (Fig. 8). Even at long distances, the binding interfaces can be pulled together. Thereby, the interaction between the P1 residue of BPTI, a lysine, and the S1 pocket of trypsin assumes a central role. Comparable to the association of barnase and barstar where charged and polar residues are the first to make contact (26), for BPTI, the P1 residue serves as anchor residue (97,98) that binds early in the binding process to the S1 pocket. It forms an ionic interaction with residue D189 at the bottom of the S1 pocket (for

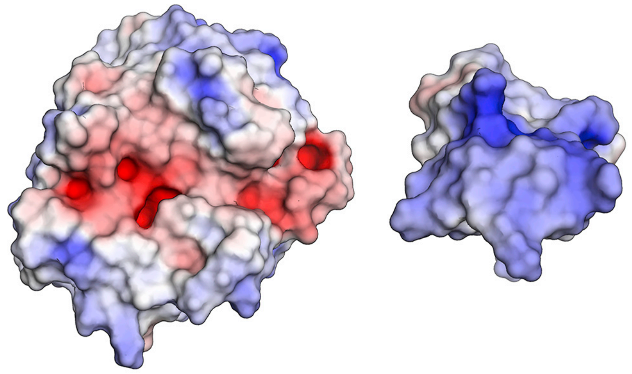

FIGURE 8 Electrostatics for trypsin and BPTI. The binding cleft of trypsin (*left*) shows negative electrostatics (*red coloring*). The binding interface of BPTI (*right*) is positively charged (*blue*). To see this figure in color, go online.

a contact map, see Fig. S14). As anchor residues hold the encounter complex in place, the rest of the binding interface has time to adjust without dissociating again. This is also in line with studies that show the effect of mutations to alanine at the binding interface of BPTI (58). Generally, these mutations increase the dissociation rate, likely by destabilizing the complex. The mutation K15A additionally lowers the association rate, which also agrees with the here presented model, as the electrostatically driven association cannot take place and the formation of an encounter complex features a higher kinetic barrier. Comparing the contacts in the encounter complex and in the native complex (Figs. S14 and S15), especially contacts of the prime site, are not yet present in the encounter complex. The formation of nonnative salt bridges, which stabilize the encounter complex as observed in binding to a PDZ domain (39), does not take place in the binding of BPTI to trypsin. Contacts that are formed in the encounter complex and are not present in the native complex include trypsin residues in the surrounding area of the binding site and can be explained by a sliding movement of BPTI along the surface (e.g., K15(P1)−G219, K15(P1)−C220, R17(P2′)–G148, R17(P2′)–T149, P13(P3)–Q175). The unbound state has considerably fewer, more transient contacts, many of which include nonprime site residues (for a list of the most frequently formed contacts refer to Table S1).

We observe that the encounter complex has already lost some of the rotational freedom of the unbound ensemble (Fig. 5). However, prime and nonprime sites are not yet optimally aligned, but still rather free to move and largely solvated. A partial loss of rotational freedom is also observed by Kozakov et al. (30) They compare the shape of the energy surface during association to a canyon because the movement possibilities are reduced to a two-dimensional subspace without large barriers, where the binding partners can slide into place. This description also matches the tilting movement that we see in Fig. 5, observable by the increasingly restricted angles between the binding sites. Although the encounter complex is structurally still rather diverse (Fig. 5), its energetics (Fig. 3) and particularly the number of water molecules surrounding the binding interface (Fig. 4) are well defined.

Both the transition from the unbound ensemble to the encounter complex and the transition from the encounter complex to the native complex are associated with the loss of a specific number of water molecules from the solvation shells of the binding interface. The expulsion of water molecules seems to be a critical factor that causes barriers in the binding process (28). However, especially at the last stage of the binding process, water molecules are displaced from hydrophobic areas at the binding interface (Fig. S16). As the proteins approach, they can essentially trap water molecules between them, whose displacement could give a negative free energy contribution and promote the last binding step.

The displacement of the water molecules allows a further approaching of the binding partners and the formation of additional and stronger interactions. In the transition to the native ensemble short-range interactions, especially van der Waals interactions (Fig. 3), increase, whereas the shape complementarity between the binding partners is essential for the perfect fit. Notably, BPTI residue R17, at the P2′ position, has to rearrange and find a conformation that fits into the prime site of trypsin. This extended horizontal conformation of R17 is imperative for the formation of the native complex. Kimura et al. (97) compare the function of R17 to a latch, which holds the proteins together in the complex. Additionally, the P1 residue, K15, has to adopt an extended conformation and an orientation to exactly fit into the S1 pocket. We find that this conformation is already present in the apo form and becomes more favorable over the course of the binding process (Fig. 7). A similar picture results for trypsin. For the protease, a conformation in which prime site residue Y39 builds the top of the S1′ pocket is preferred to a conformation where it extends toward peripheral regions of the prime site or toward the solvent in the complex. As for BPTI, the native conformation gains relevance on the way from the unbound state to the encounter complex and from the encounter complex to the native complex (Fig. 6). This observation strongly suggests conformational selection, as the binding-competent conformations are already present in the apo forms. The population shift can be directly observed in the probabilities of the clusters, which change as the binding partners approach and unsuitable, clashing conformations cannot be assumed anymore. Thereby, the conformational selection seems to take place in a stepwise manner, as the likelihood for the native conformation increases in each phase (99). During the binding, the flexibility of the binding sites and therefore the conformational entropy decreases (Fig. S17; (7,100)), as the mobility becomes more restricted.

Overall, the binding mechanism that we observe is similar to the concepts that Grünberg et al. (9) describe and is in line with our previously proposed hypothesis (20). However, in

this study, we provide a substantially more exhaustive approach, in which we explicitly trace the recognition pathway in atomistic detail. In summary, we report that the association phase is largely driven by long-ranging electrostatic interactions and ends in an encounter complex. In this stage, the binding sites are already prealigned but mostly still solvated. In the next step, water molecules are displaced from the binding interface, and the complex can form from shape-complementary conformations. Grünberg et al. characterize an additional step in which these recognition complexes refold to build the native complex. Possibly due to a low free energy barrier connected to this step, we only see one conformationally and energetically uniform recognition complex, which corresponds to the native complex. We derive this proposed two-step mechanism (schematic representation in Fig. 9) using trypsin-BPTI binding as well-studied model systems. However, our model is in line with previous findings for a broad range of protein complexes indicating a certain generalizability. We presume that the fundamental insights on protein-protein complex formation discussed in this study, will broadly benefit the design and optimization of novel biopharmaceuticals.

CONCLUSIONS

In this study, we suggest a two-step binding mechanism following the paradigm of conformational selection with a population shift. In the first step, long-range electrostatic interactions promote loose association and steer the binding partner into an advantageous orientation. The salt bridge between BPTI's P1 residue (K15) and D189 at the S1 pocket anchors the proteins and hinders them from drifting apart. In this preoriented encounter complex, the binding partners can adjust the alignment of their binding interface and select matching conformations. By displacing the solvent and assuming optimal shape complementarity, the short-range interactions are maximized, and the stable native complex can be formed. During the binding process, especially prime site residues of both trypsin and BPTI can assume a variety of conformations. In accordance with the conformational selection mechanism, the binding-competent conformations of both proteins are already present in their dynamic apo ensembles and increase in probability along the binding pathway.

A detailed understanding of protein-protein recognition is of great benefit for drug discovery efforts in the design and improvement of therapeutic biologics. Here, we provide a description of the stages of protein recognition in atomic detail. The formation of encounter complexes along binding pathways is often guided by electrostatic interactions and prearrangements of the binding partners. For the formation of the native complex, the displacement of water molecules, shape complementary, adaptability, and optimization to the interactions are vital and have strong effects on the affinity. These insights on the association pathway can be utilized to improve protein-protein docking search algorithms and scoring functions.

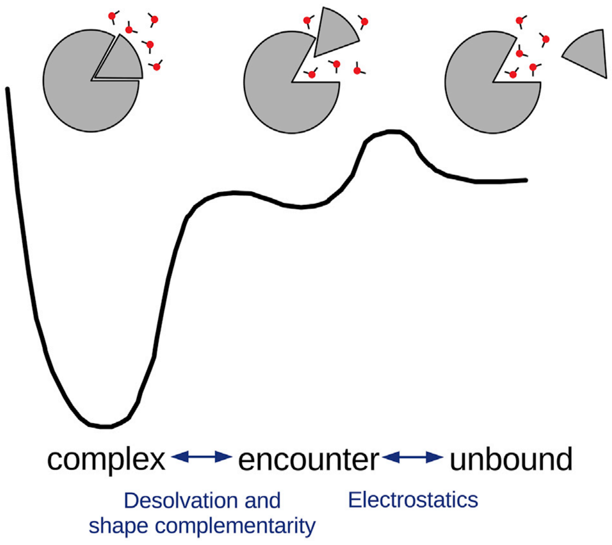

FIGURE 9 Schematic representation of the binding pathway. Electrostatic interactions are the driving force causing the formation of the encounter complex. As the binding partner further approach, the binding interface have to align, especially at the prime site, and the remaining water molecules have to be displaced to allow the optimal shape complementarity in the native complex. To see this figure in color, go online.

SUPPORTING MATERIAL

Supporting Material can be found online at https://doi.org/10.1016/j.bpj.2020.06.032.

AUTHOR CONTRIBUTIONS

U.K., A.S.K., F.W., J.K., and K.R.L. designed the research. U.K. carried out the simulations and analyzed the data. U.K. drafted the manuscript, all authors were involved in the interpretation of the data and the writing of the manuscript.

ACKNOWLEDGMENTS

The authors thank Florian Hofer, Patrick Quoika, and Alexander Spinn for their input in fruitful discussions.

Funding for this work has been provided by the Austrian Science Fund (P30565 and P30737). U.K. received a scholarship from the vice-rectorship for research of the University of Innsbruck.

REFERENCES

1. Walsh, G. 2018. Biopharmaceutical benchmarks 2018. *Nat. Biotechnol.* 36:1136–1145.
2. Fischer, E. 1894. Einfluss der Configuration auf die Wirkung der Enzyme. *Ber. Dtsch. Chem. Ges.* 27:2985–2993.
3. Koshland, D. E., Jr. 1959. Enzyme flexibility and enzyme action. *J. Cell. Comp. Physiol.* 54:245–258.

4. Ma, B., S. Kumar, …, R. Nussinov. 1999. Folding funnels and binding mechanisms. *Protein Eng.* 12:713–720.

5. Kumar, S., B. Ma, …, R. Nussinov. 2000. Folding and binding cascades: dynamic landscapes and population shifts. *Protein Sci.* 9:10–19.

6. Boehr, D. D., R. Nussinov, and P. E. Wright. 2009. The role of dynamic conformational ensembles in biomolecular recognition. *Nat. Chem. Biol.* 5:789–796.

7. Wlodarski, T., and B. Zagrovic. 2009. Conformational selection and induced fit mechanism underlie specificity in noncovalent interactions with ubiquitin. *Proc. Natl. Acad. Sci. USA.* 106:19346–19351.

8. Csermely, P., R. Palotai, and R. Nussinov. 2010. Induced fit, conformational selection and independent dynamic segments: an extended view of binding events. *Trends Biochem. Sci.* 35:539–546.

9. Grünberg, R., J. Leckner, and M. Nilges. 2004. Complementarity of structure ensembles in protein-protein binding. *Structure.* 12:2125–2136.

10. Schreiber, G. 2002. Kinetic studies of protein-protein interactions. *Curr. Opin. Struct. Biol.* 12:41–47.

11. Schreiber, G., G. Haran, and H. X. Zhou. 2009. Fundamental aspects of protein-protein association kinetics. *Chem. Rev.* 109:839–860.

12. Camacho, C. J., Z. Weng, …, C. DeLisi. 1999. Free energy landscapes of encounter complexes in protein-protein association. *Biophys. J.* 76:1166–1178.

13. Gabdoulline, R. R., and R. C. Wade. 2001. Protein-protein association: investigation of factors influencing association rates by brownian dynamics simulations. *J. Mol. Biol.* 306:1139–1155.

14. Myles, T., B. F. Le Bonniec, …, S. R. Stone. 2001. Electrostatic steering and ionic tethering in the formation of thrombin-hirudin complexes: the role of the thrombin anion-binding exosite-I. *Biochemistry.* 40:4972–4979.

15. Sinha, N., and S. J. Smith-Gill. 2002. Electrostatics in protein binding and function. *Curr. Protein Pept. Sci.* 3:601–614.

16. Suh, J. Y., C. Tang, and G. M. Clore. 2007. Role of electrostatic interactions in transient encounter complexes in protein-protein association investigated by paramagnetic relaxation enhancement. *J. Am. Chem. Soc.* 129:12954–12955.

17. Alsallaq, R., and H. X. Zhou. 2007. Energy landscape and transition state of protein-protein association. *Biophys. J.* 92:1486–1502.

18. Dagliyan, O., E. A. Proctor, …, N. V. Dokholyan. 2011. Structural and dynamic determinants of protein-peptide recognition. *Structure.* 19:1837–1845.

19. Tworowski, D., A. V. Feldman, and M. G. Safro. 2005. Electrostatic potential of aminoacyl-tRNA synthetase navigates tRNA on its pathway to the binding site. *J. Mol. Biol.* 350:866–882.

20. Waldner, B. J., J. Kraml, …, K. R. Liedl. 2018. Electrostatic recognition in substrate binding to serine proteases. *J. Mol. Recognit.* 31:e2727.

21. Batra, J., A. Szabó, …, E. S. Radisky. 2013. Long-range electrostatic complementarity governs substrate recognition by human chymotrypsin C, a key regulator of digestive enzyme activation. *J. Biol. Chem.* 288:9848–9859.

22. Frisch, C., A. R. Fersht, and G. Schreiber. 2001. Experimental assignment of the structure of the transition state for the association of barnase and barstar. *J. Mol. Biol.* 308:69–77.

23. Gray, J. J., S. Moughon, …, D. Baker. 2003. Protein-protein docking with simultaneous optimization of rigid-body displacement and side-chain conformations. *J. Mol. Biol.* 331:281–299.

24. Tang, C., J. Iwahara, and G. M. Clore. 2006. Visualization of transient encounter complexes in protein-protein association. *Nature.* 444:383–386.

25. Schilder, J., and M. Ubbink. 2013. Formation of transient protein complexes. *Curr. Opin. Struct. Biol.* 23:911–918.

26. Hoefling, M., and K. E. Gottschalk. 2010. Barnase-Barstar: from first encounter to final complex. *J. Struct. Biol.* 171:52–63.

27. Plattner, N., S. Doerr, …, F. Noé. 2017. Complete protein-protein association kinetics in atomic detail revealed by molecular dynamics simulations and Markov modelling. *Nat. Chem.* 9:1005–1011.

28. Levy, Y., and J. N. Onuchic. 2006. Water mediation in protein folding and molecular recognition. *Annu. Rev. Biophys. Biomol. Struct.* 35:389–415.

29. Kraml, J., A. S. Kamenik, …, K. R. Liedl. 2019. Solvation free energy as a measure of hydrophobicity: application to serine protease binding interfaces. *J. Chem. Theory Comput.* 15:5872–5882.

30. Kozakov, D., K. Li, …, S. Vajda. 2014. Encounter complexes and dimensionality reduction in protein-protein association. *eLife.* 3:e01370.

31. Camacho, C. J., and S. Vajda. 2001. Protein docking along smooth association pathways. *Proc. Natl. Acad. Sci. USA.* 98:10636–10641.

32. Pallara, C., M. Rueda, …, J. Fernández-Recio. 2016. Conformational heterogeneity of unbound proteins enhances recognition in protein-protein encounters. *J. Chem. Theory Comput.* 12:3236–3249.

33. Spaar, A., and V. Helms. 2005. Free energy landscape of protein-protein encounter resulting from Brownian dynamics simulations of Barnase: Barstar. *J. Chem. Theory Comput.* 1:723–736.

34. Northrup, S. H., and H. P. Erickson. 1992. Kinetics of protein-protein association explained by Brownian dynamics computer simulation. *Proc. Natl. Acad. Sci. USA.* 89:3338–3342.

35. Gabdoulline, R. R., and R. C. Wade. 2002. Biomolecular diffusional association. *Curr. Opin. Struct. Biol.* 12:204–213.

36. Ahmad, M., W. Gu, and V. Helms. 2008. Mechanism of fast peptide recognition by SH3 domains. *Angew. Chem. Int.Engl.* 47:7626–7630.

37. Chong, S. H., and S. Ham. 2012. Impact of chemical heterogeneity on protein self-assembly in water. *Proc. Natl. Acad. Sci. USA.* 109:7636–7641.

38. Schmidt, A. G., H. Xu, …, S. C. Harrison. 2013. Preconfiguration of the antigen-binding site during affinity maturation of a broadly neutralizing influenza virus antibody. *Proc. Natl. Acad. Sci. USA.* 110:264–269.

39. Blöchliger, N., M. Xu, and A. Caflisch. 2015. Peptide binding to a PDZ domain by electrostatic steering via nonnative salt bridges. *Biophys. J.* 108:2362–2370.

40. Li, Z. L., and M. Buck. 2019. Modified potential functions result in enhanced predictions of a protein complex by all-atom molecular dynamics simulations, confirming a stepwise association process for native protein-protein interactions. *J. Chem. Theory Comput.* 15:4318–4331.

41. Henzler-Wildman, K., and D. Kern. 2007. Dynamic personalities of proteins. *Nature.* 450:964–972.

42. Karplus, M., and J. A. McCammon. 2002. Molecular dynamics simulations of biomolecules. *Nat. Struct. Biol.* 9:646–652.

43. Neumann, J., and K. E. Gottschalk. 2009. The effect of different force applications on the protein-protein complex Barnase-Barstar. *Biophys. J.* 97:1687–1699.

44. Wang, L., S. W. I. Siu, …, V. Helms. 2010. Downhill binding energy surface of the barnase-barstar complex. *Biopolymers.* 93:977–985.

45. Moritsugu, K., T. Terada, and A. Kidera. 2014. Energy landscape of all-atom protein-protein interactions revealed by multiscale enhanced sampling. *PLoS Comput. Biol.* 10:e1003901.

46. Kim, Y. C., C. Tang, …, G. Hummer. 2008. Replica exchange simulations of transient encounter complexes in protein-protein association. *Proc. Natl. Acad. Sci. USA.* 105:12855–12860.

47. Kastner, J. 2011. Umbrella sampling. *Wires Comput Mol Sci.* 1:932–942.

48. Halfon, S., T. T. Baird, and C. S. Craik. 2004. Trypsin. *In* Handbook of Proteolytic Enzymes. A. J. Barrett, N. D. Rawlings, and J. F. Woessner, eds. Elsevier Ltd, pp. 1483–1488.

49. Rawlings, N. D., A. J. Barrett, …, R. D. Finn. 2018. The MEROPS database of proteolytic enzymes, their substrates and inhibitors in

2017 and a comparison with peptidases in the PANTHER database. *Nucleic Acids Res.* 46:D624–D632.

50. Schechter, I., and A. Berger. 1967. Protease subsite nomenclature. *Biochem. Biophys. Res. Commun.* 27:157–162.
51. Buch, I., T. Giorgino, and G. De Fabritiis. 2011. Complete reconstruction of an enzyme-inhibitor binding process by molecular dynamics simulations. *Proc. Natl. Acad. Sci. USA.* 108:10184–10189.
52. Plattner, N., and F. Noé. 2015. Protein conformational plasticity and complex ligand-binding kinetics explored by atomistic simulations and Markov models. *Nat. Commun.* 6:7653.
53. Huber, R., D. Kukla, …, H. Formanek. 1970. The basic trypsin inhibitor of bovine pancreas. I. Structure analysis and conformation of the polypeptide chain. *Naturwissenschaften.* 57:389–392.
54. Wlodawer, A., J. Walter, …, L. Sjölin. 1984. Structure of bovine pancreatic trypsin inhibitor. Results of joint neutron and X-ray refinement of crystal form II. *J. Mol. Biol.* 180:301–329.
55. Wagner, G., A. DeMarco, and K. Wüthrich. 1976. Dynamics of the aromatic amino acid residues in the globular conformation of the basic pancreatic trypsin inhibitor (BPTI). I. 1H NMR studies. *Biophys. Struct. Mech.* 2:139–158.
56. Shaw, D. E., P. Maragakis, …, W. Wriggers. 2010. Atomic-level characterization of the structural dynamics of proteins. *Science.* 330:341–346.
57. Vincent, J. P., and M. Lazdunski. 1972. Trypsin-pancreatic trypsin inhibitor association. Dynamics of the interaction and role of disulfide bridges. *Biochemistry.* 11:2967–2977.
58. Castro, M. J. M., and S. Anderson. 1996. Alanine point-mutations in the reactive region of bovine pancreatic trypsin inhibitor: effects on the kinetics and thermodynamics of binding to beta-trypsin and alpha-chymotrypsin. *Biochemistry.* 35:11435–11446.
59. Krowarsch, D., M. Dadlez, …, J. Otlewski. 1999. Interscaffolding additivity: binding of P1 variants of bovine pancreatic trypsin inhibitor to four serine proteases. *J. Mol. Biol.* 289:175–186.
60. Brandsdal, B. O., J. Aqvist, and A. O. Smalås. 2001. Computational analysis of binding of P1 variants to trypsin. *Protein Sci.* 10:1584–1595.
61. Brandsdal, B. O., and A. O. Smalås. 2000. Evaluation of protein-protein association energies by free energy perturbation calculations. *Protein Eng.* 13:239–245.
62. Sadiq, S. K., F. Noé, and G. De Fabritiis. 2012. Kinetic characterization of the critical step in HIV-1 protease maturation. *Proc. Natl. Acad. Sci. USA.* 109:20449–20454.
63. Chodera, J. D., and F. Noé. 2014. Markov state models of biomolecular conformational dynamics. *Curr. Opin. Struct. Biol.* 25:135–144.
64. Shukla, D., Y. Meng, …, V. S. Pande. 2014. Activation pathway of Src kinase reveals intermediate states as targets for drug design. *Nat. Commun.* 5:3397.
65. Sun, X., S. Singh, …, G. R. Bowman. 2018. Simulation of spontaneous G protein activation reveals a new intermediate driving GDP unbinding. *eLife.* 7:e38465.
66. Kawamura, K., T. Yamada, …, N. Niimura. 2011. X-ray and neutron protein crystallographic analysis of the trypsin-BPTI complex. *Acta Crystallogr. D Biol. Crystallogr.* 67:140–148.
67. Marquart, M., J. Walter, …, R. Huber. 1983. The geometry of the reactive site and of the peptide groups in trypsin, trypsinogen and its complexes with inhibitors. *Acta Crystallogr. B.* 39:480–490.
68. Molecular Operating Environment (MOE): Chemical Computing Group Inc., Montreal, QC, Canada.
69. Labute, P. 2009. Protonate3D: assignment of ionization states and hydrogen coordinates to macromolecular structures. *Proteins.* 75:187–205.
70. Jorgensen, W. L., J. Chandrasekhar, …, M. L. Klein. 1983. Comparison of simple potential functions for simulating liquid water. *J. Chem. Phys.* 79:926–935.
71. Case, D. A., I. Y. Ben-Shalom, …, P. A. Kollman. 2018. AMBER 2018. University of California, San Francisco, CA.
72. Wallnoefer, H. G., S. Handschuh, …, T. Fox. 2010. Stabilizing of a globular protein by a highly complex water network: a molecular dynamics simulation study on factor Xa. *J. Phys. Chem. B.* 114:7405–7412.
73. Salomon-Ferrer, R., A. W. Götz, …, R. C. Walker. 2013. Routine microsecond molecular dynamics simulations with AMBER on GPUs. 2. Explicit solvent particle mesh Ewald. *J. Chem. Theory Comput.* 9:3878–3888.
74. Maier, J. A., C. Martinez, …, C. Simmerling. 2015. ff14SB: improving the accuracy of protein side chain and backbone parameters from ff99SB. *J. Chem. Theory Comput.* 11:3696–3713.
75. Darden, T., D. York, and L. Pedersen. 1993. Particle mesh Ewald: an N•log(N) method for Ewald sums in large systems. *J. Chem. Phys.* 98:10089–10092.
76. Adelman, S. A. 1976. Quantum generalized Langevin equation approach to gas/solid collisions. *Chem. Phys. Lett.* 40:495–499.
77. Berendsen, H. J. C., J. P. M. Postma, …, J. R. Haak. 1984. Molecular-dynamics with coupling to an external bath. *J. Chem. Phys.* 81:3684–3690.
78. Ciccotti, G., and J. P. Ryckaert. 1986. Molecular dynamics simulation of rigid molecules. *Comput. Phys. Rep.* 4:345–392.
79. Kumar, S., D. Bouzida, …, J. M. Rosenberg. 1992. The weighted histogram analysis method for free-energy calculations on biomolecules. 1. The method. *J. Comput. Chem.* 13:1011–1021.
80. Scherer, M. K., B. Trendelkamp-Schroer, …, F. Noé. 2015. PyEMMA 2: a software package for estimation, validation, and analysis of Markov models. *J. Chem. Theory Comput.* 11:5525–5542.
81. Shao, J., S. W. Tanner, …, T. E. Cheatham. 2007. Clustering molecular dynamics trajectories: 1. Characterizing the performance of different clustering algorithms. *J. Chem. Theory Comput.* 3:2312–2334.
82. Pérez-Hernández, G., F. Paul, …, F. Noé. 2013. Identification of slow molecular order parameters for Markov model construction. *J. Chem. Phys.* 139:015102.
83. Schwantes, C. R., and V. S. Pande. 2013. Improvements in Markov state model construction reveal many non-native interactions in the folding of NTL9. *J. Chem. Theory Comput.* 9:2000–2009.
84. Prinz, J. H., H. Wu, …, F. Noé. 2011. Markov models of molecular kinetics: generation and validation. *J. Chem. Phys.* 134:174105.
85. Husic, B. E., and V. S. Pande. 2018. Markov state models: from an art to a science. *J. Am. Chem. Soc.* 140:2386–2396.
86. Roblitz, S., and M. Weber. 2013. Fuzzy spectral clustering by PCCA plus: application to Markov state models and data classification. *Adv. Data Anal. Classif.* 7:147–179.
87. Noé, F., C. Schütte, …, T. R. Weikl. 2009. Constructing the equilibrium ensemble of folding pathways from short off-equilibrium simulations. *Proc. Natl. Acad. Sci. USA.* 106:19011–19016.
88. The PyMOL Molecular Graphics System: Schrödinger LLC, New York.
89. Jurrus, E., D. Engel, …, N. A. Baker. 2018. Improvements to the APBS biomolecular solvation software suite. *Protein Sci.* 27:112–128.
90. Nguyen, C. N., T. K. Young, and M. K. Gilson. 2012. Grid inhomogeneous solvation theory: hydration structure and thermodynamics of the miniature receptor cucurbit[7]uril. *J. Chem. Phys.* 137:044101.
91. Nguyen, C. N., A. Cruz, …, T. Kurtzman. 2014. Thermodynamics of water in an enzyme active site: grid-based hydration analysis of coagulation factor xa. *J. Chem. Theory Comput.* 10:2769–2780.
92. Biswas, M., B. Lickert, and G. Stock. 2018. Metadynamics enhanced Markov modeling of protein dynamics. *J. Phys. Chem. B.* 122:5508–5514.
93. Fernández-Quintero, M. L., J. R. Loeffler, …, K. R. Liedl. 2019. Characterizing the diversity of the CDR-H3 loop conformational

ensembles in relationship to antibody binding properties. *Front. Immunol.* 9:3065.

94. Kahler, U., A. S. Kamenik, …, K. R. Liedl. 2020. Sodium-induced population shift drives activation of thrombin. *Sci. Rep.* 10:1086.

95. Hedstrom, L. 2002. Serine protease mechanism and specificity. *Chem. Rev.* 102:4501–4524.

96. Wehmeyer, C., M. K. Scherer, …, F. Noé. 2019. Introduction to Markov state modeling with the PyEMMA software—v1. 0. *Living J. Comput. Mol. Sci.* 1:5965.

97. Kimura, S. R., R. C. Brower, …, C. J. Camacho. 2001. Dynamical view of the positions of key side chains in protein-protein recognition. *Biophys. J.* 80:635–642.

98. Rajamani, D., S. Thiel, …, C. J. Camacho. 2004. Anchor residues in protein-protein interactions. *Proc. Natl. Acad. Sci. USA.* 101:11287–11292.

99. Ma, B., and R. Nussinov. 2010. Enzyme dynamics point to stepwise conformational selection in catalysis. *Curr. Opin. Chem. Biol.* 14:652–659.

100. Fuchs, J. E., R. G. Huber, …, K. R. Liedl. 2015. Dynamics govern specificity of a protein-protein interface: substrate recognition by thrombin. *PLoS One.* 10:e0140713.

Biophysical Journal, Volume *119*

Supplemental Information

Protein-Protein Binding as a Two-Step Mechanism: Preselection of Encounter Poses during the Binding of BPTI and Trypsin

Ursula Kahler, Anna S. Kamenik, Franz Waibl, Johannes Kraml, and Klaus R. Liedl

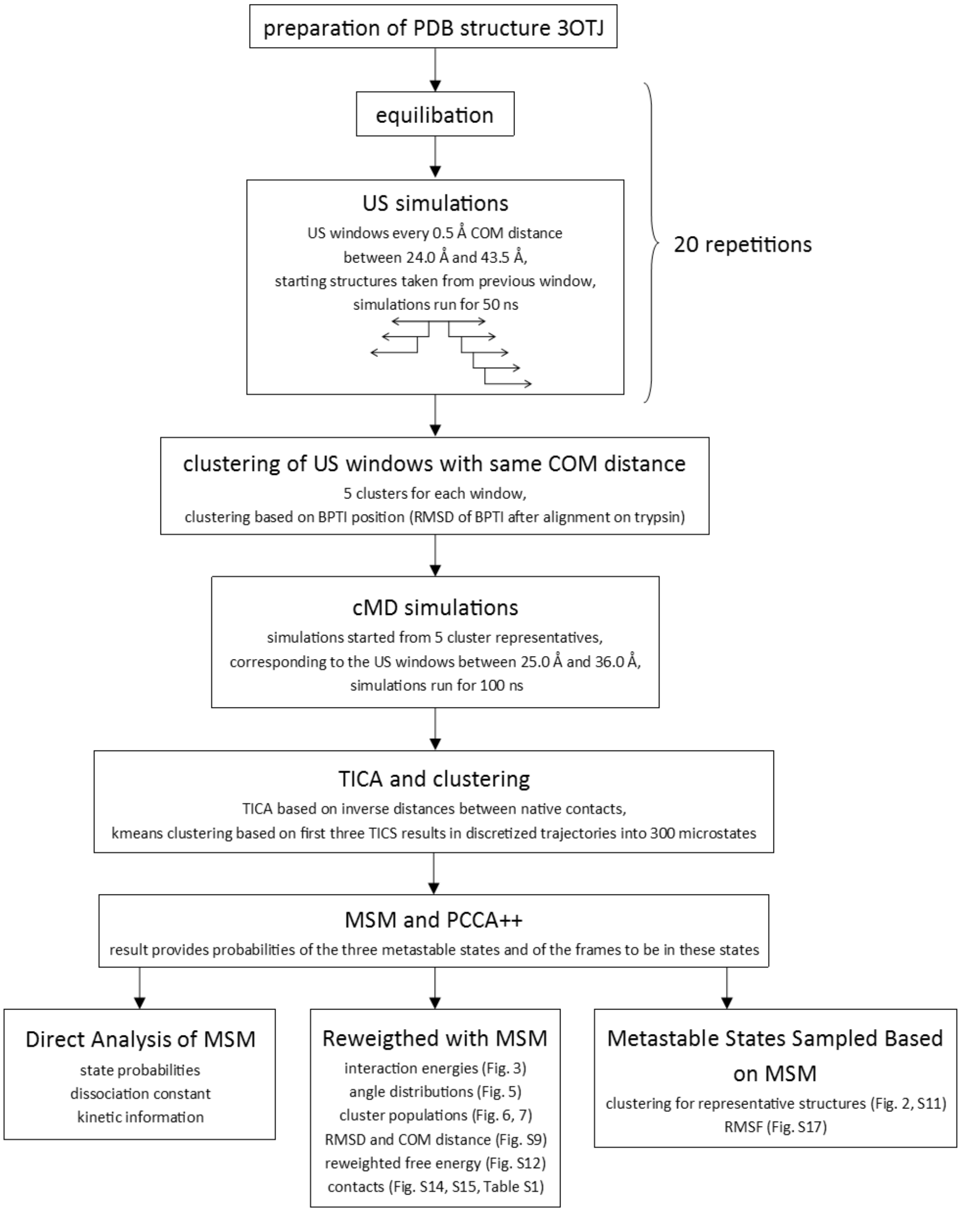

Figure S1: Workflow applied in the preparation of this paper. After preparation and equilibation of a crystal structure of the Trypsin–BPTI complex, twenty separate US simulations are used to sample the unbinding. As these cover the whole pathway, it is expected that using them to seed cMD simulations strongly enhances the sampling efficiency. Based on the cMD simulations, an MSM is build following the typical workflow, including coordinate reduction and discretization of the trajectories. The MSM determines the classification into three metastable states and builds the foundation for the subsequent analyses.

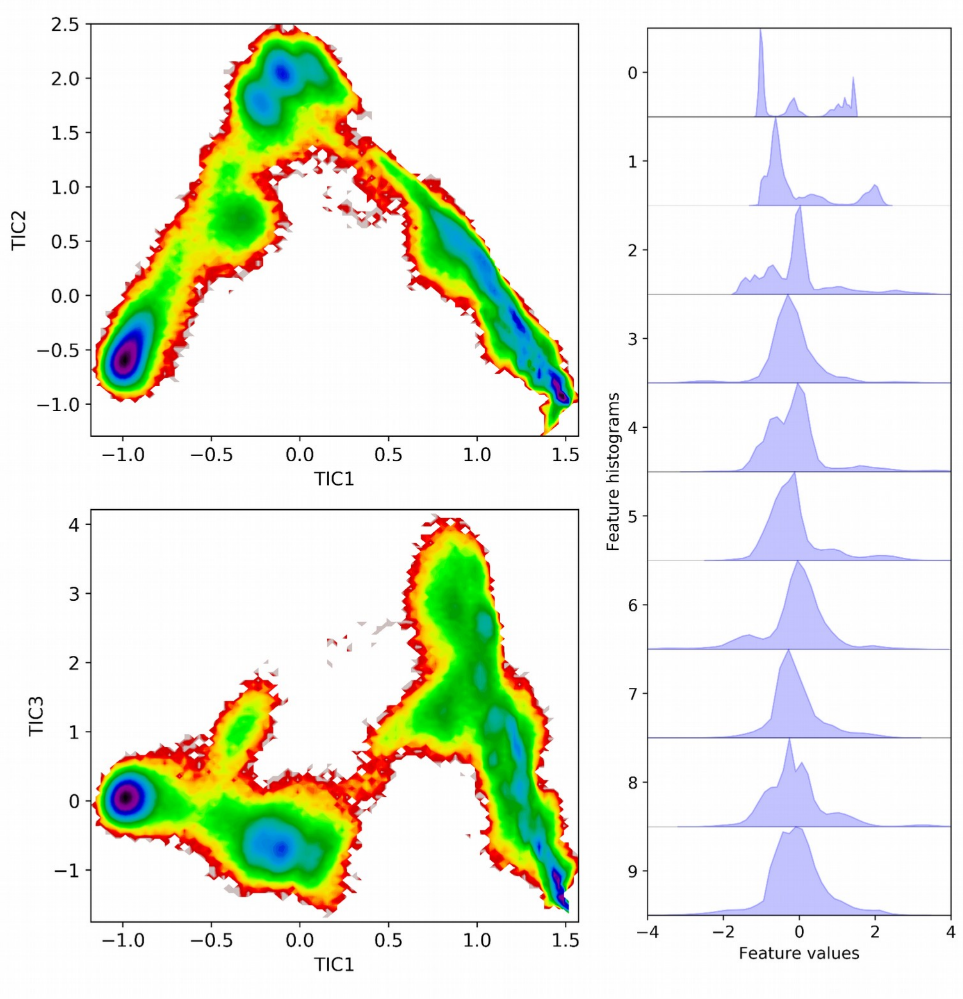

Figure S2: Projection of the simulation frames on the TICs. Top left: Density of the frames within TIC1 and TIC2; bottom left: density of the frames within TIC1 and TIC3; right: distribution of the frames within the first ten TICs.

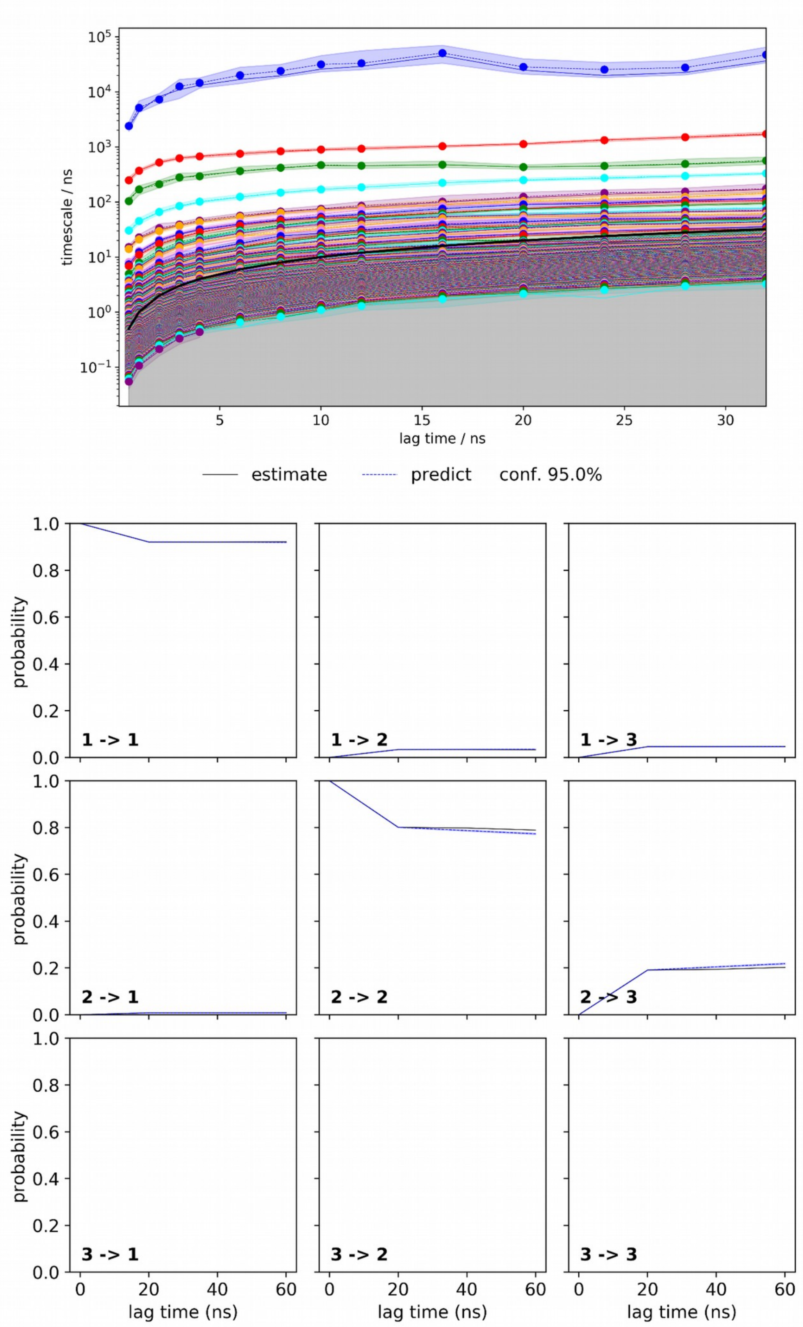

Figure S3: Chapman-Kolmogorov tests of the MSM. Top: The implied timescale plot helps to choose the lag time, for which the calculated timescales are constant. We chose a lag time of 20 ns although the loss of connectivity causes deviations from the ideal behavior. Bottom: The plots show the result of the Chapman-Kolmogorov test for 20, 40 and 60 ns, comparing the results predicted by the used MSM (lag time of 20 ns, dashed line) and estimated at the different lag times (continuous line). In some panels, both lines can be hardly seen, as they run along the 0- (2 -> 1, 3 -> 1 and 3 -> 2) or 1-line (3 -> 3) of the plots. Thereby, 1 denotes the unbound state, 2 the encounter state and 3 the complex state.

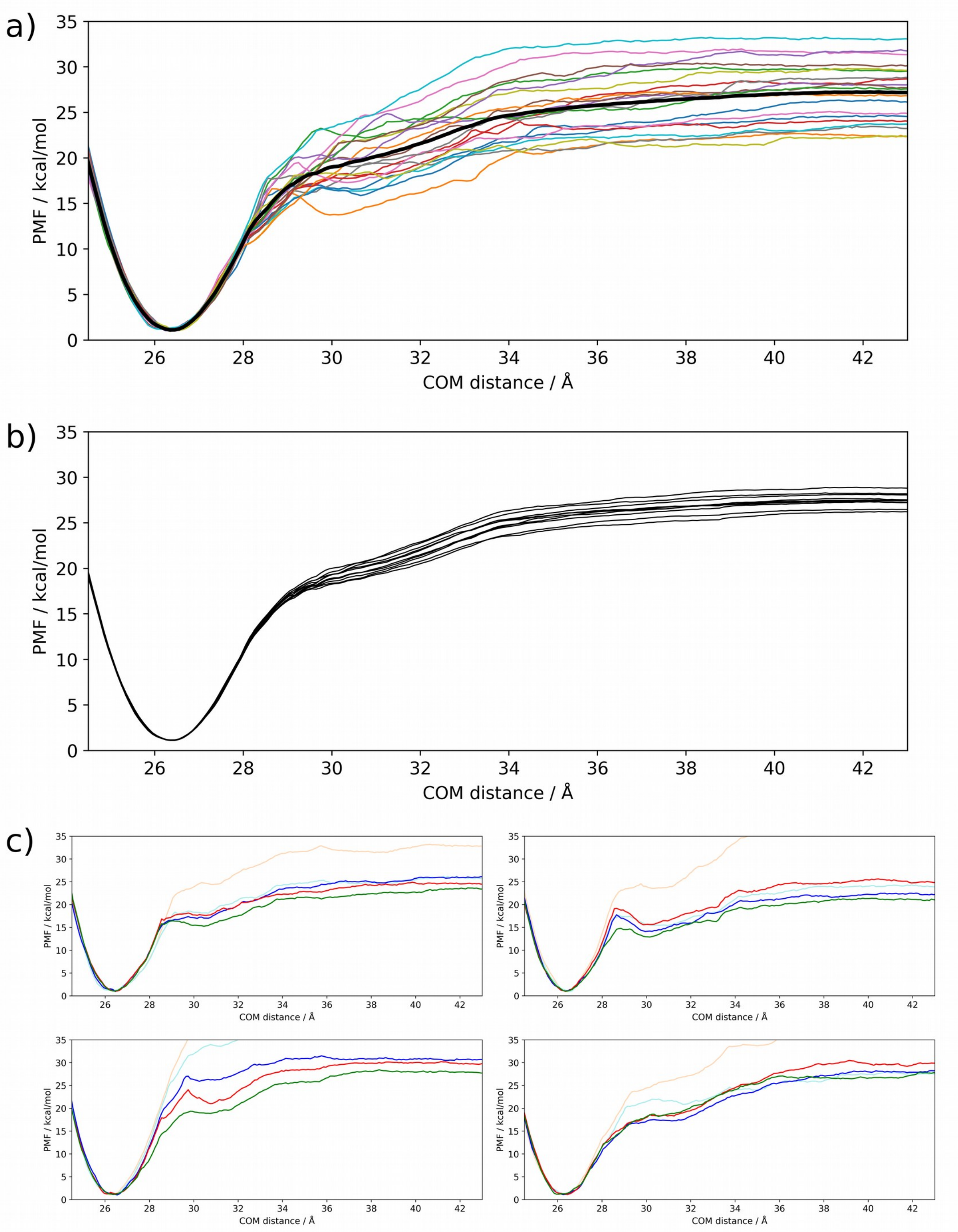

Figure S4: PMF curves of the US runs. a) The PMF curves vary for the 20 runs (colored lines). The PMF calculated from the combined runs is shown as black line. b) 10 bootstrapping resamples of the US runs have been produced by randomly combining the different US runs. c) To assess the convergence of the US runs, the US windows are splitted into 10 ns segments and the PMF is calculated for these segments separately. The pastel colors (pink, light blue) represent the segments of 0–10 ns and 10–20 ns respectively, which have been removed for the clustering and analysis of the simulations. The blue (20–30 ns), red (30–40 ns) and green lines (40–50 ns) show the PMF for the other segments. The result is shown for four runs.

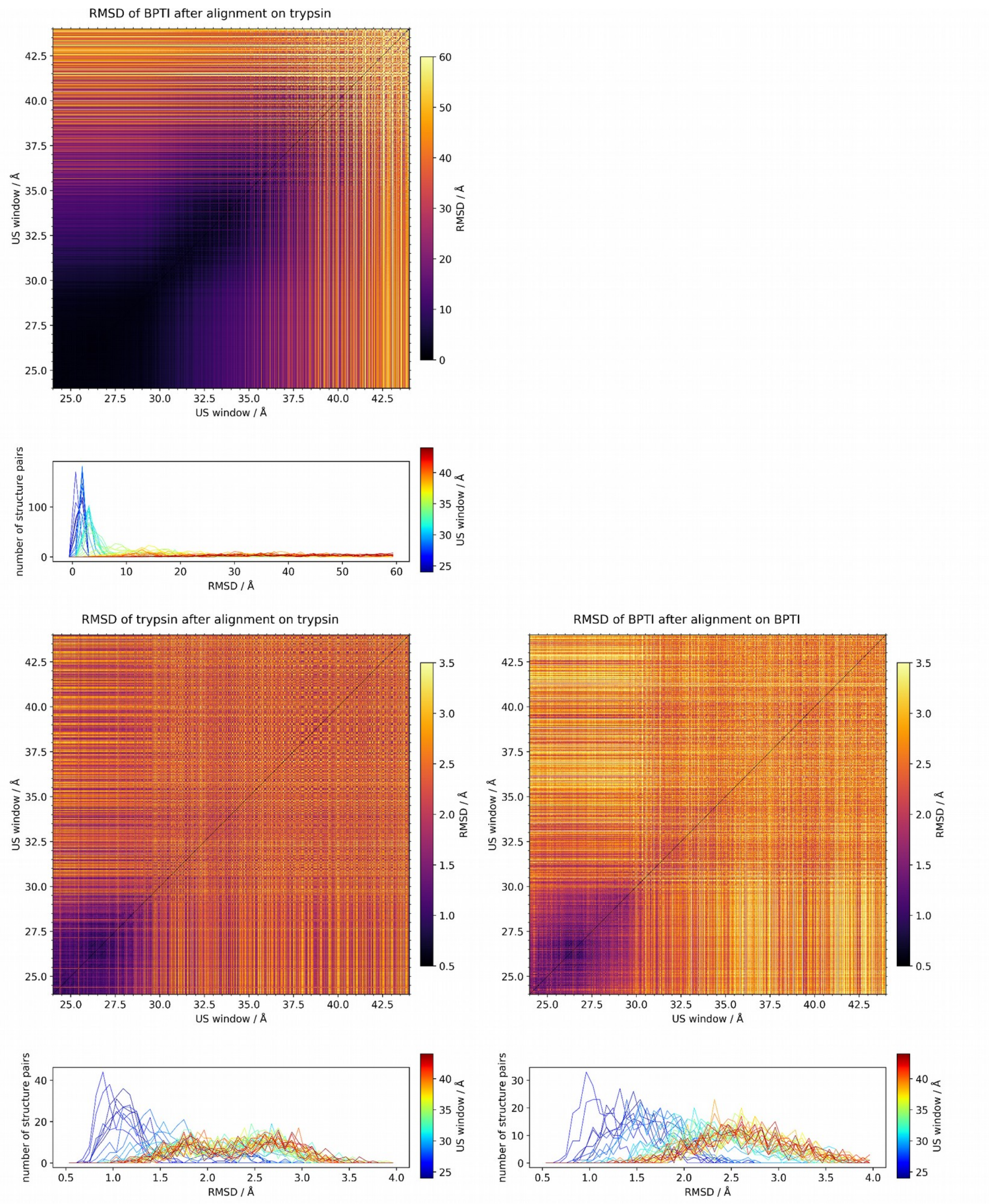

Figure S5: RMSD between the starting structures of the windows of all 20 US runs. The RMSD is calculated of BPTI after alignment on trypsin (top), of trypsin after alignment on trypsin (bottom left) and of BPTI after alignment on BPTI (bottom right). 2D-RMSD plots show the RMSD between all starting structures, sorted by the COM distance of the US window. Additionally, the distributions of RMSD values between starting structures belonging to the single COM distances are shown. On the one hand, the RMSD plots reveal the variety of BPTI positions. As unbinding takes place, an increasing number of different BPTI positions appear in the starting structures. At the same time, the internal conformations do not vary strongly, as internal degrees of freedom are not boosted by the US. However, it is clearly visible that the starting structures at small COM distances are very similar to each other and more varying at the US windows with larger distances.

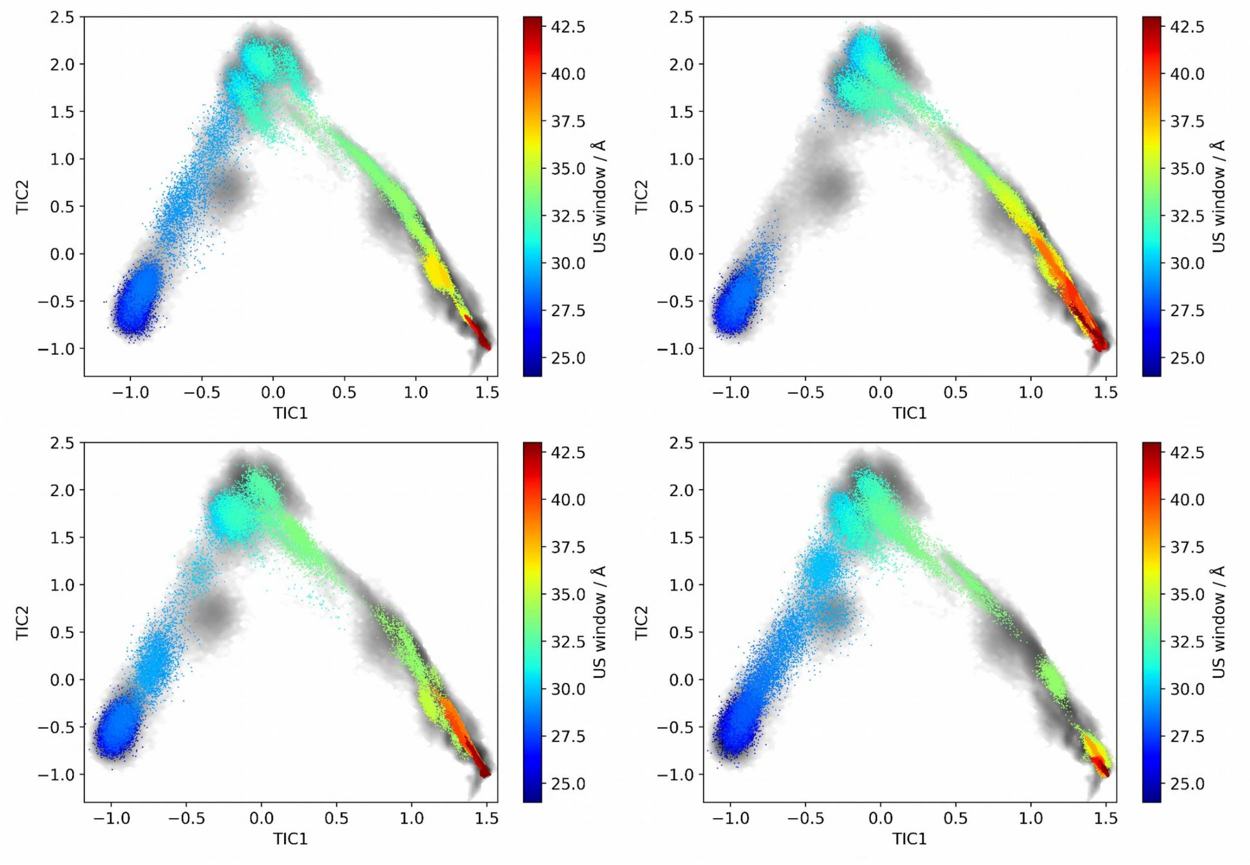

Figure S6: Projection of US runs into the TICA space. Roughly the same conformational space is occupied by the US runs (colored) as by the cMD simulations (grey) on which the TICA is based. Not all local minima are covered by all runs, suggesting that the sampled pathways vary. The result for four of the twenty runs is shown.

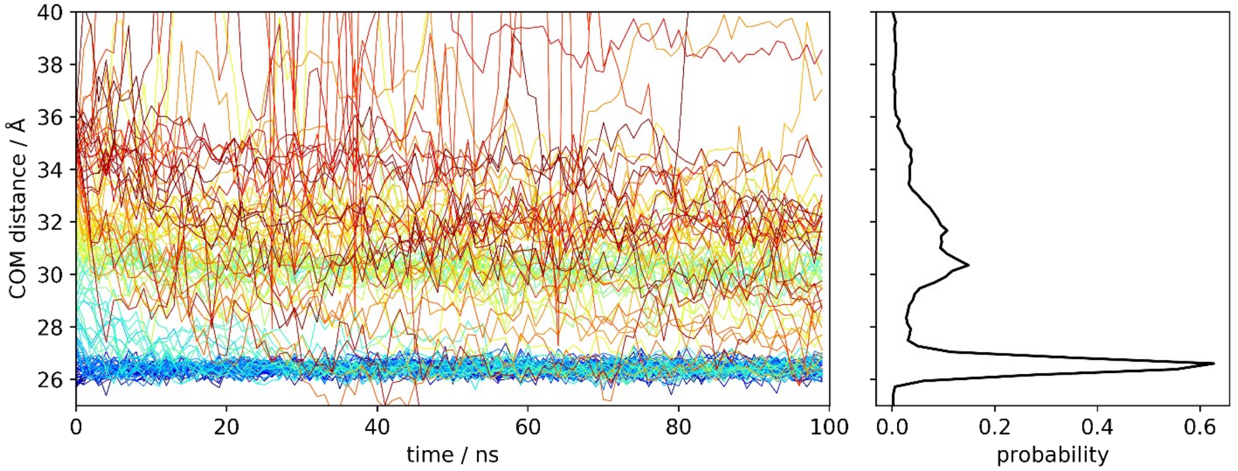

Figure S7: The progress of the cMD simulations started from conformations with different BPTI positions. The colors of the lines indicate from which umbrella window the starting structures originate, from dark blue being the starting structures from the smallest used CV window (25.0 Å) to dark red from the largest used CV window (36.0 Å). The panel on the right side shows the distribution of COM distances in these simulations.

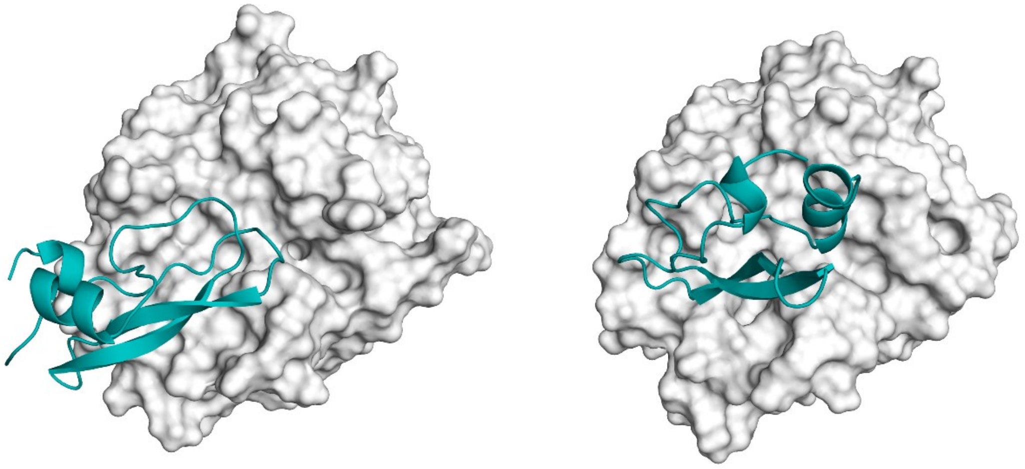

Figure S8: Representative structures, where BPTI has a flat orientation on the surface of trypsin. They lead to small (i.e., smaller than in the X-ray) COM distances. The structures were generated by clustering BPTI positions (alignment on trypsin), where the COM distance is smaller than 25.0 Å.

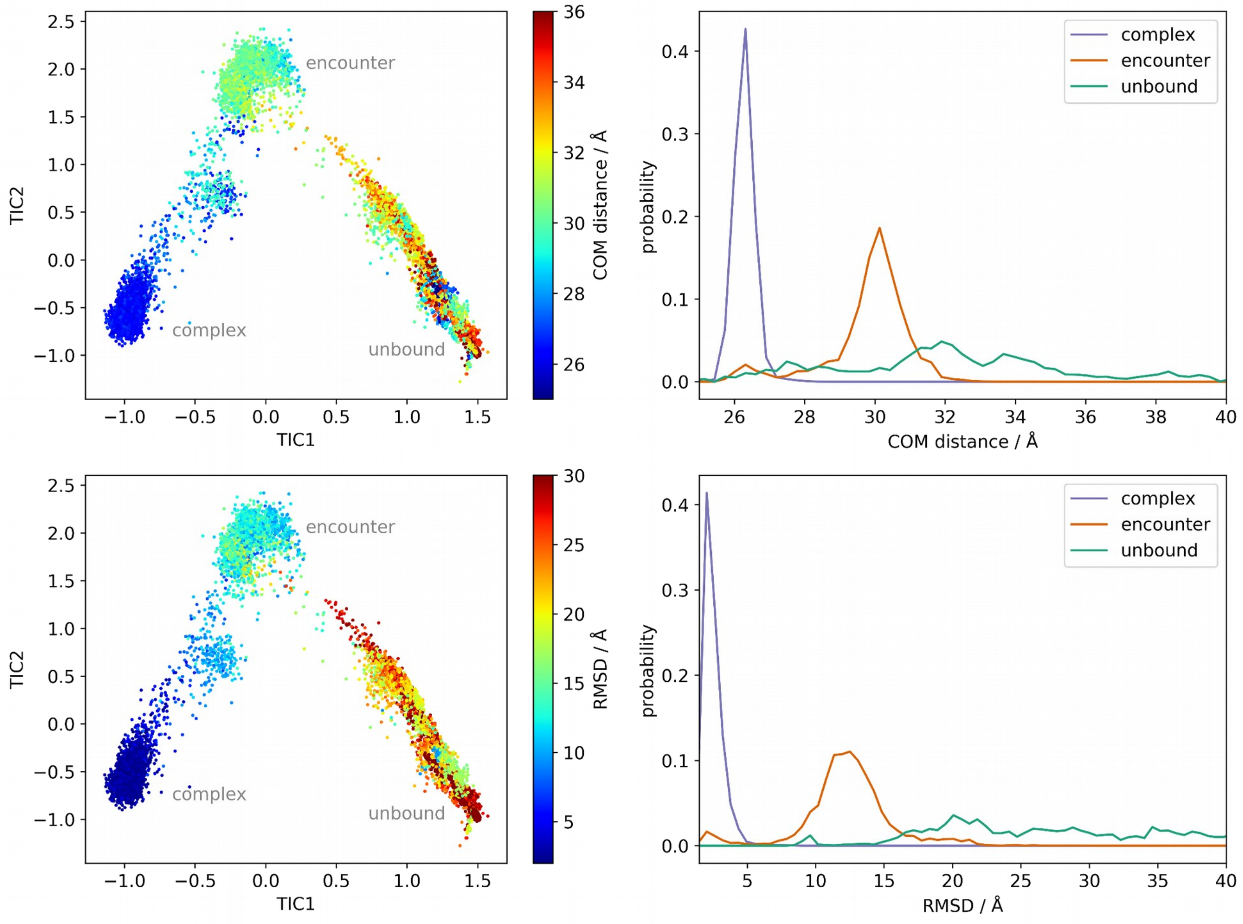

Figure S9: Distance of BPTI in cMD simulations. Left. The frames are projected onto the TICA space and colored according to the BPTI distance. Right: The distribution within the complex, encounter and unbound state is shown, as calculated from the MSM. Top: The distance between the centers of mass of trypsin and BPTI does not determine the state classification, e.g., in the unbound state very short distances can occur as BPTI can loosely associate to areas remote from the native binding site. Bottom: The RMSD values of BPTI (alignment on trypsin) in respect to its X-ray pose show that in the ensemble of the native complex, all configureations are similar to the X-ray structure, while in the unbound state, the vast majority of the positions of BPTI differ strongly from the native position.

TIC1

Ranking	Trypsin residue	BPTI residue	Maximal correla-tion of atom pair
1	W215	C14(P2)	-0.972
2	Q192	A16(P1’)	-0.970
3	Q192	K15(P1)	-0.969
4	C191	K15(P1)	-0.967
5	S214	C14(P2)	-0.965
6	S214	K15(P1)	-0.965
7	G193	A16(P1’)	-0.964
8	S190	K15(P1)	-0.963
9	D194	K15(P1)	-0.960
10	V213	K15(P1)	-0.959
11	S195	K15(P1)	-0.958
12	G193	K15(P1)	-0.957

TIC2

Ranking	Trypsin residue	BPTI residue	Maximal correla-tion of atom pair
1	G219	K15(P1)	0.559
2	C220	K15(P1)	0.501
3	F41	R17(P2’)	-0.406
4	H40	R17(P2’)	-0.386
5	C191	K15(P1)	0.380
6	G193	R17(P2’)	-0.372
7	W215	P13(P3)	0.370
8	Q192	T11(P5)	-0.370
9	Y39	I19(P4’)	-0.368
10	Y39	R17(P2’)	-0.361
11	F41	I19(P4’)	-0.359
12	S195	K15(P1)	-0.347

TIC3

Ranking	Trypsin residue	BPTI residue	Maximal correla-tion of atom pair
1	W215	P13(P3)	0.347
2	L99	P13(P3)	0.330
3	G216	P13(P3)	0.285
4	W215	K15(P1)	0.251
5	L99	C14(P2)	0.230
6	Q192	K15(P1)	0.203
7	S217	P13(P3)	0.197
8	L99	C38	0.193
9	Q192	G12(P4)	0.190
10	N97	R39	0.187
11	T98	R39	0.185
12	L99	R39	0.184

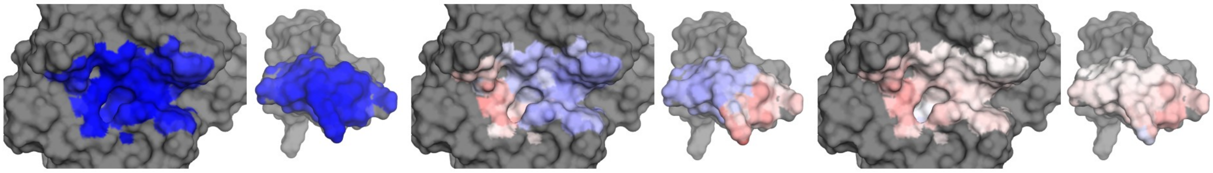

Figure S10: Correlation between the inverse distances of native atom pairs and the TICs. The table lists the largest correlations (according to absolute values) for TIC1, TIC2 and TIC3. It takes the largest correlation for each residue pair and neglects all following atom pairs of the same residues. (Clearly, neighboring atoms often show similar correlations, however to list them does not bear much additional information.) Underneath, each residue is colored according to the largest correlation, with which it correlates with the TIC, from -1 (blue), over 0 (white), to 1 (red).

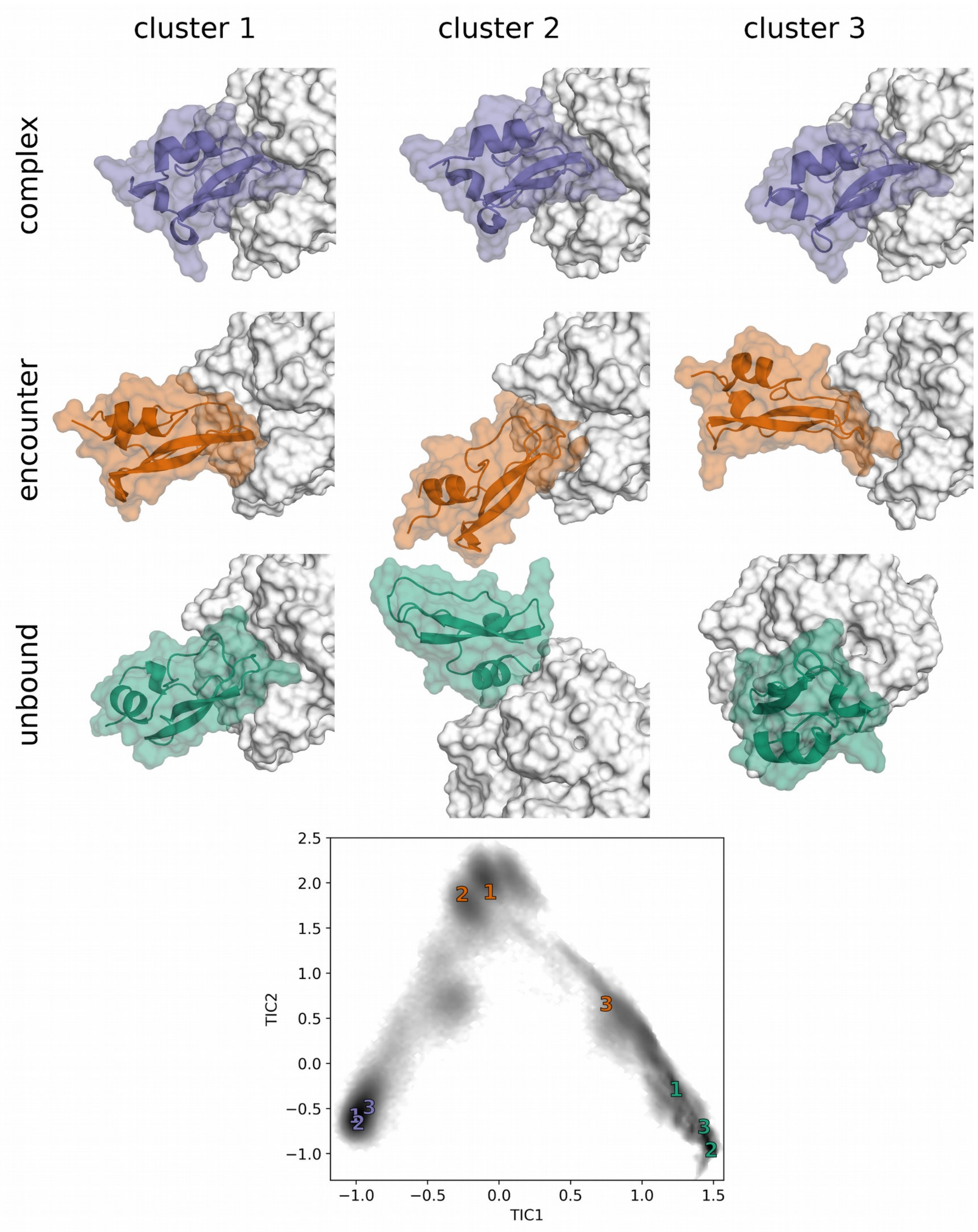

Figure S11: Representative structures of the complex (violet), encounter (orange) and unbound (green) state. Representative BPTI positions (RMSD of BPTI as cluster distance after alignment on trypsin) of the three most populated clusters are shown. The positions of these conformation in the TICA space are marked with the cluster numbers in the bottom panel.

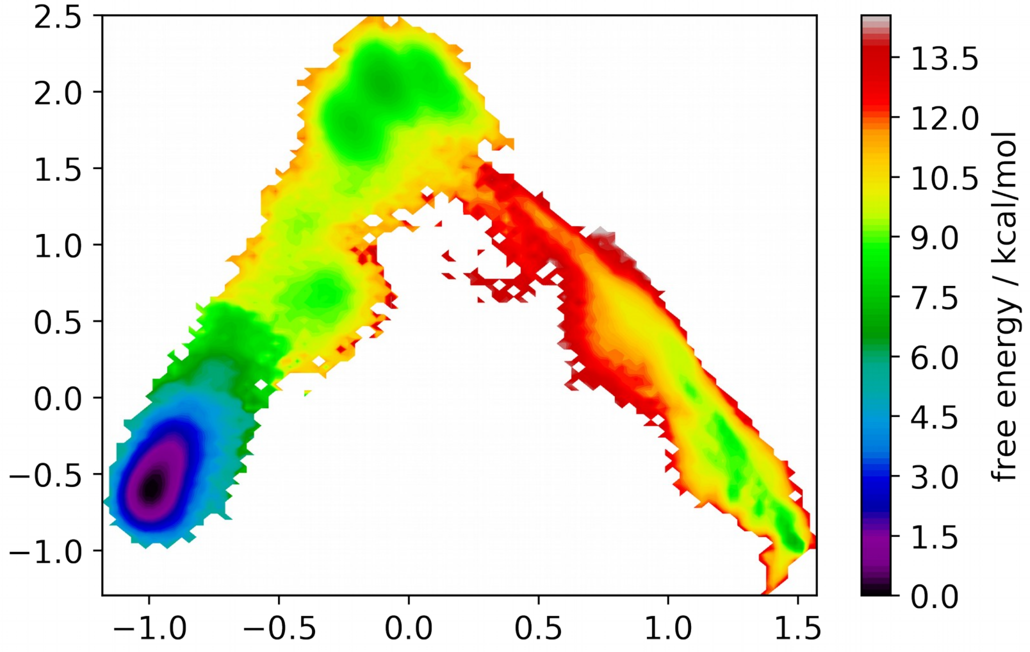

Figure S12: Reweighted free energy from MSM. The probability of the microstates calculated from the MSM has been used to reweight the distribution of the frames in the TICA space, resulting in a projection of the free energy.

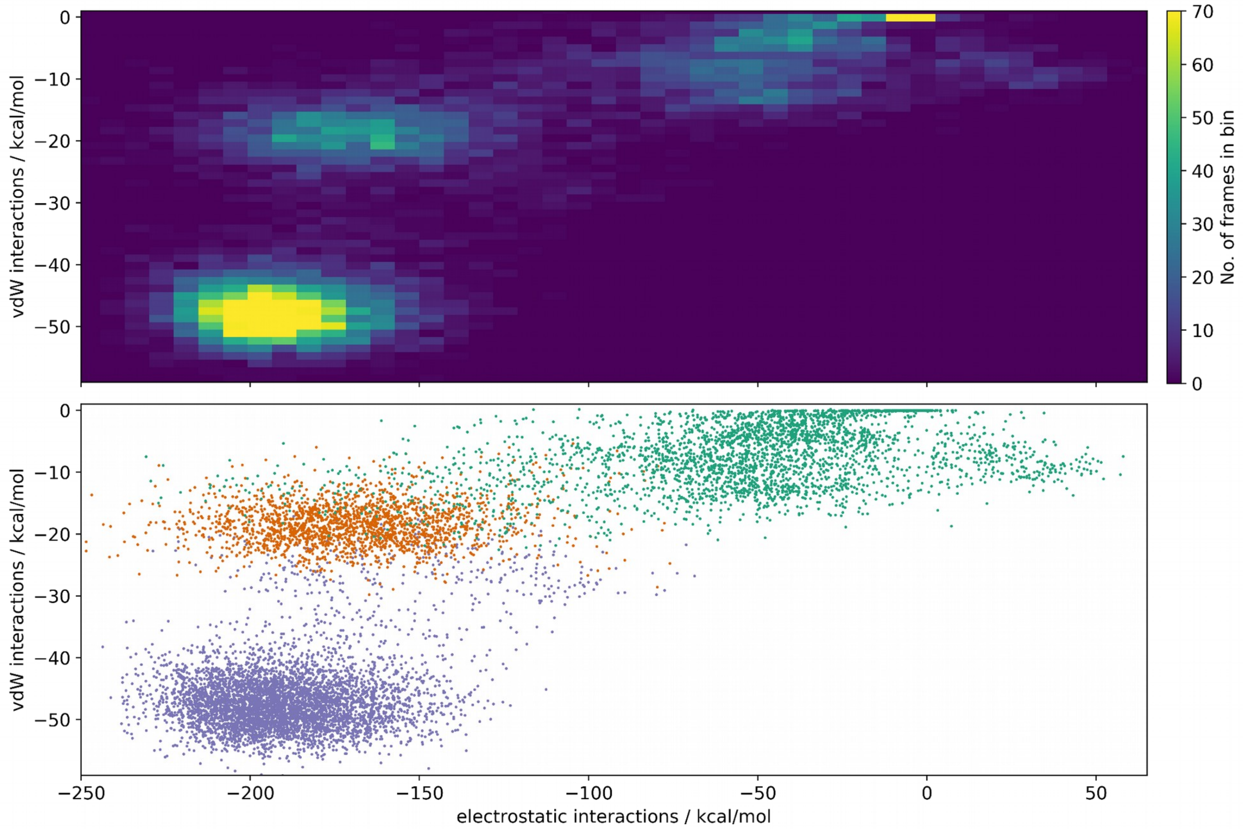

Figure S13: Van-der-Waals interactions and electrostatic interactions between the binding interface of BPTI and trypsin. Top: 2D-histogram of electrostatic and van-der-Waals interactions. Three major maxima in density can made out. Bottom: The position of the frames in the interaction space shows that the interactions mirror in the categorization into complex (violet), encounter (orange) and unbound (green) states.

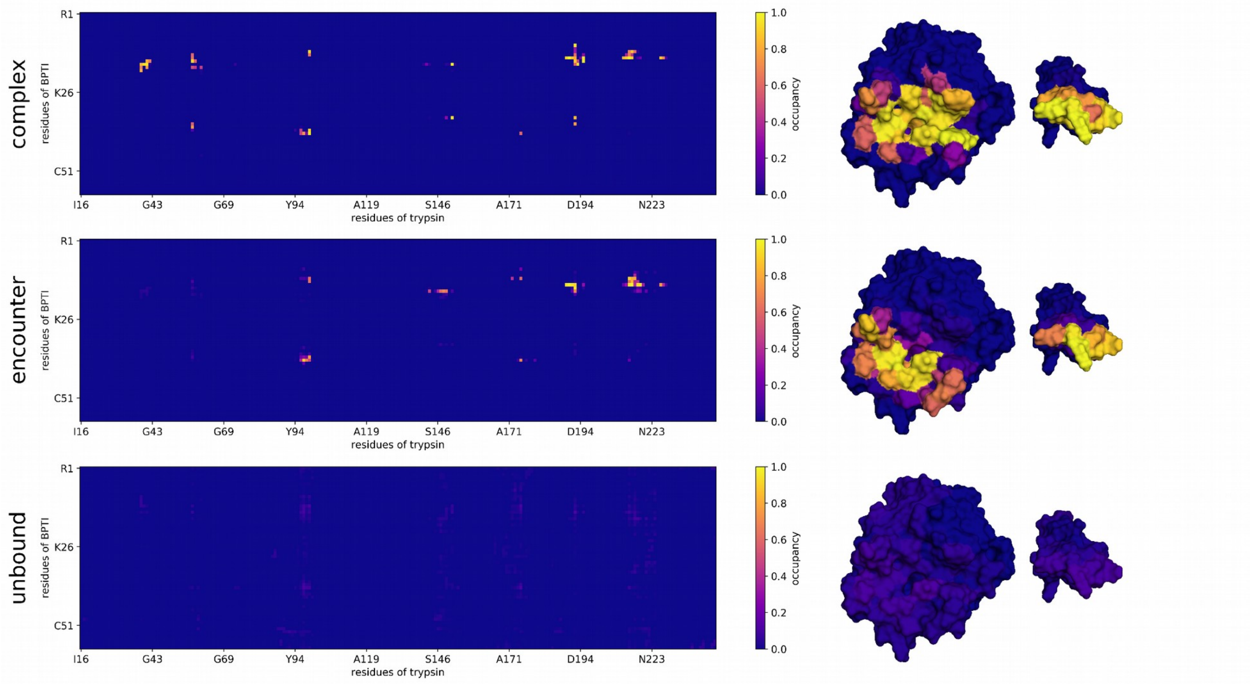

Figure S14: Contacts between trypsin and BPTI. The weighting of the contacts according to the MSM results in the division into the complex (top), encounter (middle) and unbound (bottom) state. Right: Coloring of the protein residue according to most stable contact, in which they are involved.

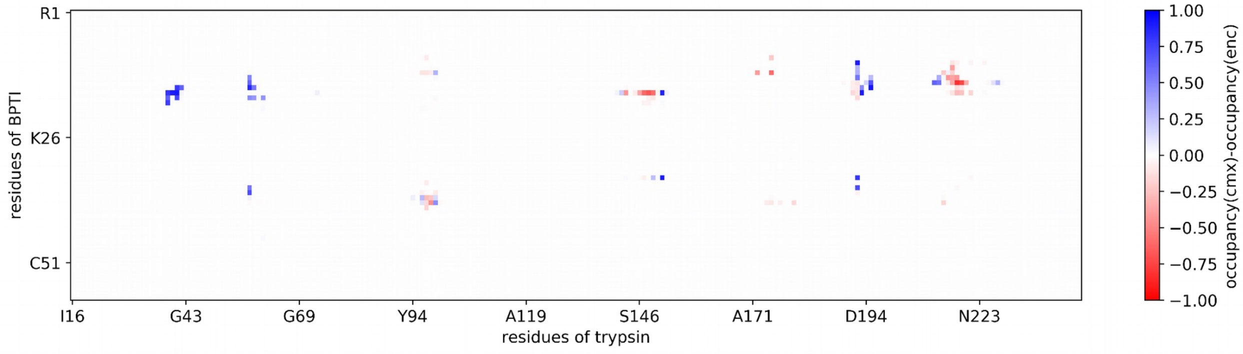

Figure S15: Difference in the residue-wise contacts between the complex and the encounter state. Blue coloring denotes contacts that have a higher occupancy in the complex state, red coloring residues that have a higher occupancy in the encounter state.

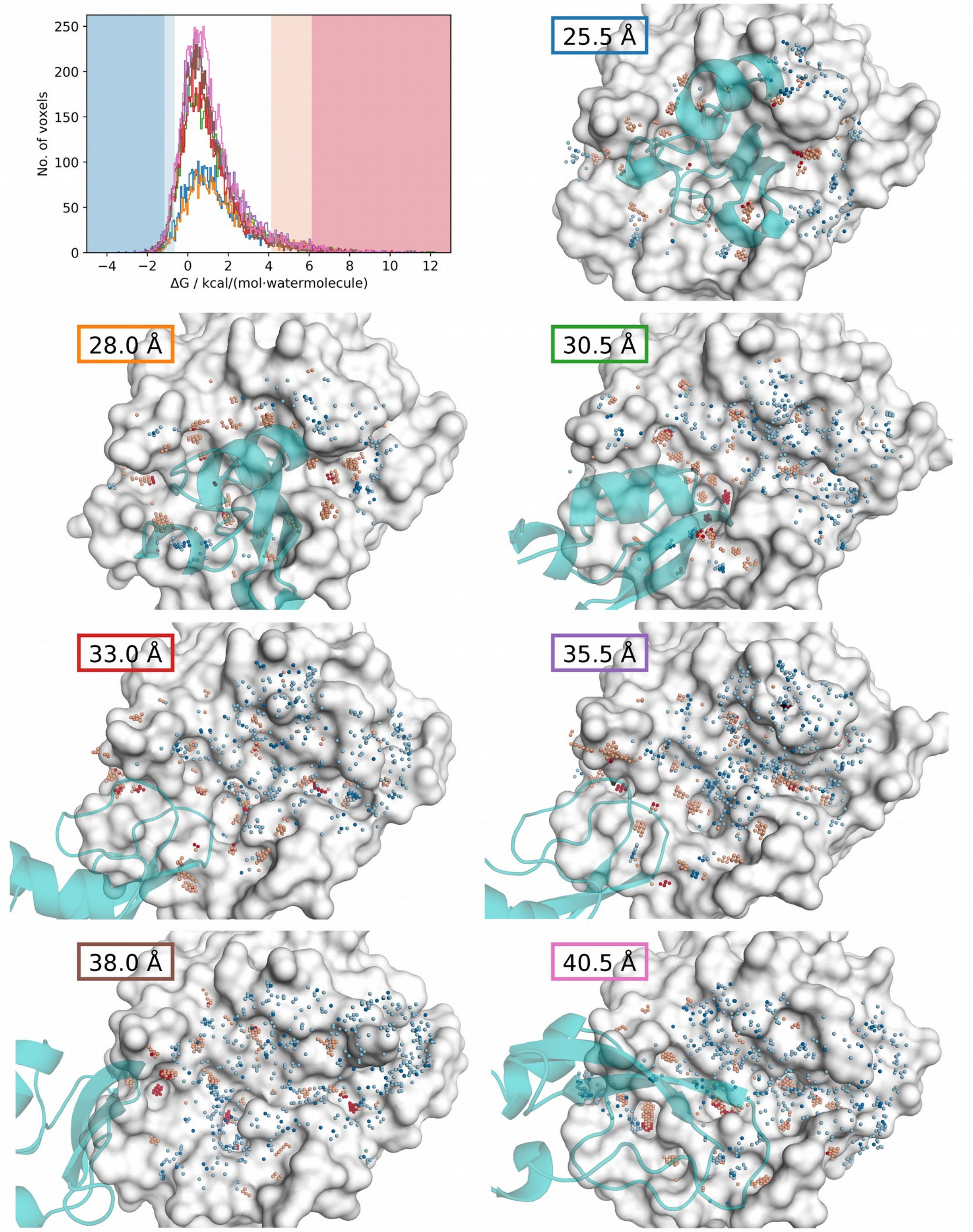

Figure S16: Free energy contributions of water molecules at the binding interface. Free energy contributions at grid points near the binding interface of trypsin have been calculated with GIST as described in the Methods section of the main document. The top-left panel shows a histogram of the free energy of water molecules at voxels with a significant water density (greater or equal to that of the bulk) at the different COM distances. The indicated 1st (blue), 5th (light blue), 95th (orange) and 99th (red) percentiles are used to restrict the shown voxel positions. The voxel positions are shown on the structures used for GIST and colored according to the free energy (red, orange, high free energy; blue, light-blue: low free energy).

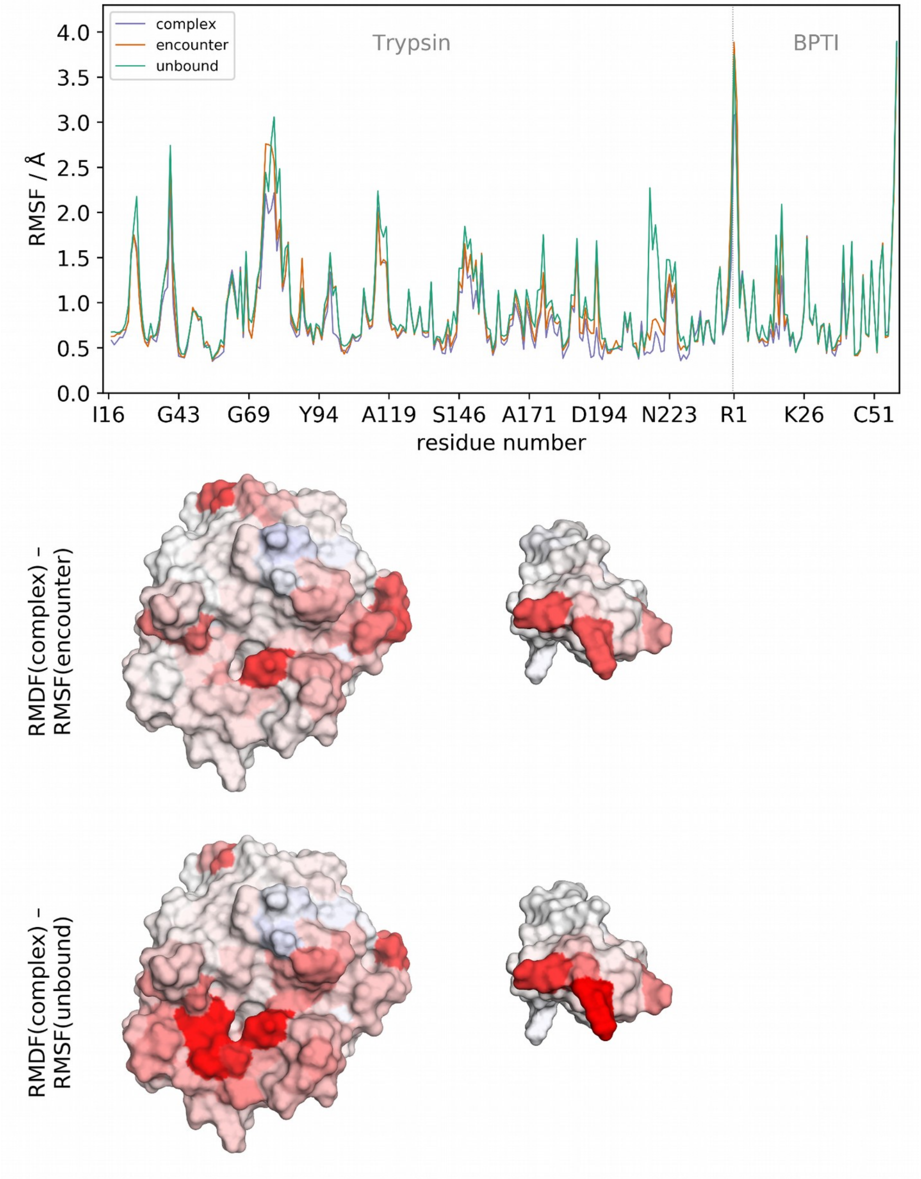

Figure S17: Residue-wise RMDF values within complex, encounter and unbound state. Top: RMSF values for trypsin and BPTI, middle: difference between the residue-wise RMDF values between complex and encounter, bottom: difference between complex and unbound from blue (-1 Å) to red (+1 Å). The calculation of the RMSF is calculated from 10,000 frames for each metastable state sampled based on the MSM.

Table S1: The 20 most highly populated contacts in the complex, encounter and unbound state. The frames have been weighted with the state probabilities from the MSM for the calculation of the occupancies.

Complex			Encounter			Unbound		
Trypsin	BPTI	occ.	Trypsin	BPTI	occ.	Trypsin	BPTI	occ.
Y151	R17	0.996	S190	K15	0.989	Q192	K15	0.123
S190	K15	0.994	G216	K15	0.987	Q175	R39	0.116
S195	K15	0.983	W215	K15	0.962	N97	R39	0.111
W215	K15	0.971	Q192	A16	0.953	Q175	P13	0.110
S195	A16	0.969	C220	K15	0.948	L99	P13	0.108
L99	R39	0.968	D189	K15	0.946	N97	K15	0.103
F41	R17	0.957	C191	K15	0.946	G219	R17	0.100
Q192	T11	0.953	G219	K15	0.934	S217	R17	0.100
G193	R17	0.949	Q192	K15	0.923	N97	C14	0.099
H40	R17	0.940	W215	P13	0.912	N97	C38	0.095
Y151	V34	0.938	N97	R39	0.892	N97	P13	0.091
H57	A16	0.929	G216	C14	0.832	W215	K15	0.086
L99	P13	0.925	S217	P13	0.812	T98	R39	0.085
D189	K15	0.910	G216	P13	0.806	L99	R39	0.084
L99	C38	0.909	L99	C38	0.765	Q175	C14	0.082
G216	K15	0.900	G226	K15	0.756	S96	K15	0.076
S214	K15	0.888	Q175	R39	0.697	Q221	R17	0.073
C191	K15	0.879	G148	R17	0.694	P92	R53	0.073
G226	K15	0.876	Q192	R17	0.686	H57	K15	0.072
Q192	A16	0.874	T98	R39	0.668	Y151	K15	0.071

Movie S1: Unbinding as sampled by an US run. BPTI is colored according to the RMSD to its native position, with green signifying a small RMSD and red a large RMSD. To obtain a continuous trajectory, only the first 10 ns of each window were used to make the video (which are not yet converged and not included in other analysis).

Movie S2: Example for a binding and unbinding event. Based on the MSM, a binding event (start at the unbound state and reaching the complex state) and an unbinding event (start at the complex state and reaching the unbound state) have been resampled. BPTI is colored according to the most probable metastable state to which the structure belongs to. The projection on the TICA space helps to understand the processes and to interpret the TICs.
